# Determinants of physical activity in community-dwelling older adults: an umbrella review

**DOI:** 10.1186/s12966-023-01528-9

**Published:** 2023-11-21

**Authors:** Cassandra D’Amore, Stephanie Saunders, Neera Bhatnagar, Lauren E. Griffith, Julie Richardson, Marla K. Beauchamp

**Affiliations:** 1 School Rehabilitation Science, McMaster University, 175 Longwood Rd South - Suite 310A, Hamilton, ON L8P 0A1 Canada; 2 Health Science Library, McMaster University, 1280 Main St W, Hamilton, ON L8S 4L8 Canada; 3 Health Research Methods, Evidence, and Impact, McMaster Univeristy, 175 Longwood Rd South - Suite 309A, Hamilton, ON L8P 0A1 Canada; 4 School of Rehabilitation Science, McMaster University, 1400 Main Street West, Institute for Applied Health Sciences (IAHS) Building - Room 403, Hamilton, ON L8S 1C7 Canada

**Keywords:** Active transport, Aging, Determinant, Factor, Overview of reviews, Physical activity, Walking

## Abstract

**Introduction:**

Physical activity (PA) is critical for disease prevention and maintaining functional ability with aging. Despite this, as many as 50% of older adults in populations worldwide are considered insufficiently active. There is a recognized need to mobilize policies targeted toward modifiable determinants of healthy aging like PA. This umbrella review aimed to summarize the evidence for determinants of PA in community-dwelling older adults.

**Methods:**

A research librarian searched six databases. Systematic and scoping reviews were included if they investigated community-dwelling people with a mean age of 60 + years and examined a relationship between a determinant and any type of PA. Two independent reviewers screened and extracted data from all reviews. JBI methodology and Critical Appraisal Checklist for Systematic Reviews and Research Syntheses were followed and information on the quality of the evidence was extracted.

**Results:**

From 17,277 records screened,11 reviews representing > 300 unique primary papers were ultimately included. Only 6% of studies included in all reviews had longitudinal designs. Included studies used a large variety of PA measures, with 76% using only self-report, 15% using only direct measures (e.g., accelerometry), 3% using both types, and 6% with no outcome measure reported. Only four reviews provided a definition of PA and there was substantial inconsistency in the way PA was categorised. Community level influences, which only included the physical environment, were the most commonly assessed (6/11) with more than 70% of the summarized relationships demonstrating null associations. Three out of four reviews reported a positive relationship between walkability and PA in general community-dwelling older adults. There was also evidence supporting relationships between presence of social support for PA, younger age, and men having higher PA from a single systematic review. None of the included reviews assessed the quality of evidence but over 60% performed a risk of bias assessment.

**Conclusions:**

Walkability, age, gender, and social support for PA were the most supported PA determinants identified. Further research should focus on interpersonal and intrapersonal influences and incorporate direct measures of PA with clear operational definitions. There is a need for longitudinal study designs to further understand determinants of PA behaviour trajectories.

**Supplementary Information:**

The online version contains supplementary material available at 10.1186/s12966-023-01528-9.

## Introduction

Physical activity (PA) is a modifiable determinant of healthy aging that promotes functional abilty [1, 2]. Physical activity has been shown to positively influence many different adverse outcomes associated with aging, including dementia [3], multi-morbidity [4], and mortality [5]. A recent estimate of public health care costs from new non-communicable disease cases associated with insufficient levels of PA found the cost of doing nothing to be 47.6 billion per year between now and 2030 [6]. Improving PA levels worldwide would not only save hundreds of billions of dollars by the end of the decade but also improve the well-being of the billions of people living into older ages.

The prevalence of physical inactivity (i.e., not meeting recommended levels of moderate-to-vigorous PA a week) has stayed the same or, in some Western countries, worsened over the last decade [7]. Globally, 25% of adults are not meeting recommended levels [8]. This trend is present across age groups but is more pronounced in older adults [9]. In line with the evidence demonstrating the importance of PA, the World Health Organization (WHO) released a global action plan to address the ongoing physical inactivity crisis through policy implementation [10]. Unfortunately, a recent update showed limited progress in increasing PA levels by 2030 [8]. In 2022, physical inactivity rates in the United States for people 70 years and older were 47% and 65% for males and females, respectively, similar to the United Kingdom (males 47% and females 56%) [11]. The lack of improvement demonstrates that a better understanding of what influences PA behaviour is essential for implementing effective policies.

Theoretical models such as the Socioecological model (SEM) demonstrate the importance of considering multiple levels of influence, including intrapersonal, interpersonal, organization, community, and policy [12, 13]. Given the large volume of research and existing systematic reviews on PA in these areas, a number of umbrella reviews examining PA determinants in different populations have been recently conducted, including in children, adults, and mixed-age groups [14–18]. However, older adults tend to favour lighter intensity PA behaviour and their motivation may differ from younger populations [19, 20]. A number of systematic reviews have focused on influences of PA behaviour in older adults but to date, no synthesis has been made to summarize these findings. Synthesizing this evidence will provide critical information for health behaviour promotion. By facilitating the presentation of evidence across reviews and within levels of influence, consistency and gaps in the literature in this area can be highlighted [21]. Therefore, the aim of this study was to conduct an umbrella review to synthesize the evidence on determinants of PA in community-dwelling older adults. Where possible evidence for determinants was examined based on type of PA outcome measure (e.g., self-report vs. direct measurement), study design (e.g., cross-sectional vs longitudinal), and sex. These data are critical to identify specific actionable steps needed to progress our understanding of the influences of PA in older adults.

## Methods

The JBI guidelines were followed for this umbrella review and where applicable, PRISMA guidelines were also used to guide reporting [21, 22]. The protocol for this umbrella review was registered with PROSPERO (CRD42020159332) and published elsewhere [23]. Terminology for this umbrella review is as follows: the term “review” will refer to the studies included in this review (i.e., systematic reviews), the terms “primary paper” or “study” will refer to the papers included in each of the included reviews (e.g., observational, experimental studies).

### Search strategy and study selection

Following preliminary searches, unique search strategies were created and carried out by NB, a McMaster University research librarian. Key terms used to create the search strategies were older adults, physical activity, systematic review, and determinant. The following databases were searched to April 2020, Cochrane Database of Systematic Reviews, CINAHL (EBSCO), MEDLINE (Ovid), Embase (Ovid), PsycINFO (Ovid), and AgeLine (EBSCO). An updated search was run in MEDLINE (Ovid) to capture any publications since the original search (included up to August 2022); search strategy available in in Table S1. Search results were organized using EndNote [24] and uploaded to Covidence [25] for screening. At least two independent reviewers piloted and screened all title and abstracts as well as full-text articles (CD, SS, and AB). Conflicts were resolved through discussion or with input from a senior team member (MB). All included reviews were hand searched for additional relevant citations. For titles and abstracts in any language other than English Google Translate was used to assess relevancy; if moved to the full text screening stage the team would seek outside assistance to assess eligibility and extract as appropriate [26, 27].

### Inclusion criteria

Reviews were included if they focused on older adults, defined as 60 years or older, or if the included studies all reported mean ages of 60 years or older. Reviews were also included if they presented a synthesis on older adults that we could extract separately (e.g., subgroup analysis). An additional criterion was added to include only reviews of community-dwelling older adults rather than any context (e.g., inpatient or assisted living) as was originally stated in the protocol. This change was made as the heterogeneity in population setting was theorised to have a large influence on the relevancy of potential determinants. Reviews were included if they contained a PA outcome that met the WHO’s definition of PA, “any bodily movement produced by skeletal muscle that requires energy expenditure” [28]. The outcome of interest for this umbrella review was relationships between determinants and PA. No restrictions were placed on the type of determinant examined. All types of measures (e.g., self-report or direct measures) for both determinants and PA were included to capture the greatest breadth of current literature. Finally, papers must have used a systematic review, scoping review, or meta-analysis methodology.

### Assessment of methodological quality and quality of the evidence

In accordance with JBI guidelines, the JBI’s Critical Appraisal Checklist for Systematic Reviews and Research Syntheses was used to assess the quality of included work [21]. The AMSTAR2 was used to determine the criteria for indicating a “yes”, “no”, “unclear”, or not applicable, for the 11 items on JBI’s critical appraisal checklist (mapping reported in Table S2) [21, 29]. Two independent reviewers (CD and SS) assessed each included review and met to discuss any conflicts. While originally planned, a formal assessment of the quality of evidence using the Grading of Recommendations, Assessment, Development and Evaluation (GRADE) was not conducted as there remains no established consensus in applying this assessment tool at the level of a review. Instead, reviewers extracted whether the quality of the evidence was assessed in each review and we considered concepts relevant to GRADE (e.g., heterogeneity, indirectness) in the summarized literature.

### Corrected covered area

The corrected covered area (CCA) was calculated to measure the overlap in primary papers among included reviews. We looked at overall CCA for this umbrella review as well as for each determinant where multiple reviews were summarized. Ratings were determined using the scale proposed by Pieper et al. [30] available in Table 1.

**Table 1:** Corrected covered area

$\mathrm{CCA}=\frac{N-r}{rc-r}$	Slight < 5%
	Moderate 5 – 9.9%
	High 10—14.9%
	Very high 15 + %

*N* - the sum of the number of primary papers in each review;* r* - total number of primary papers included (unique); *c* - number of reviews

### Data collection and summary

Two independent reviewers (CD and SS) extracted all data from reviews (i.e., did not use primary papers), and conflicts were resolved through discussion. Frequency and proportions were used to describe the review and primary paper characteristics. Determinants were grouped into broader categories where appropriate. Physical environment determinants were grouped into seven determinant categories using the Neighborhood Environment Walkability Scale (NEWS) for consistency as it was used in three of the six reviews examining the environment [31]. All category groupings can be found in Tables S3, S4 and S5. To prevent miscategorising PA types, we used a general/all PA outcome to capture everything because of the heterogeneity in PA categories used by reviews. However, we decided to group adherence to PA programs separately, as the determinants examined for this outcome were specific to exercise programs and not generalizable to other PA types (e.g., program frequency or instructor type).

#### Individual study relationships reported in each review

Relationships between determinants and PA were summarized using vote counting (i.e., comparing the number of effects favouring each direction), a synthesis method in the Cochrane handbook [32], and followed the methods outlined by Sallis et al. [33] and adapted by Choi et al. [14] for use in umbrella reviews. The number of primary studies that found a statistically significant positive, negative, or null relationship were summed for each relationship present across each review (i.e., determinant-PA combination). When a relationship was assessed more than four times within a review, the evidence was categorized as + *Cor* when > 60% of relationships were positively related, *-Cor* when > 60% of relationships were negatively related, *Null* when > 60% of relationships were not significantly related, or *IC* (i.e., inconsistent results when none of the previous conditions were met). When a relationship was examined less than four times the nomenclature *Cor* was exchanged for *Lim* (i.e., + Lim, -Lim, Null Lim) to denote limited evidence. A summary direction was provided for relationships based on the direction found for each review in the same population. The summary direction represented the majority of directions for included reviews (i.e., > 50%). If no majority was present the summary was noted as inconsistent (*IC)*.

Some of the primary studies presented results for the same relationship multiple times. Duplicates were grouped into two categories: i) two different PA outcome measures or different determinant outcome measures were used (e.g., frequency of walking and age and the amount of walking and age), and ii) the results were presented for different subgroups or moderators (e.g., age or buffer zones). For category i) each relationship was treated as unique (given a value of 1); for category ii) we followed the methods employed by Cerin et al. and applied fractional weights (all the results added to 1) [34].

#### Pooled analyses

For results presented using some form of meta-analytic approach, the details were extracted (e.g., sample size, effect estimates, direction, significance). For each review the number of effect estimates that were positive, negative or null was totalled for each relationship (i.e., a frequency count for each direction). Again, a summary direction for each relationship was provided based on the direction favoured by the majority (i.e., > 50%) of reviews. A review was said to favour a direction for pooled analyses if 60% or more of the pooled effects were in the same direction.

## Results

### Characteristics of included reviews

**Fig. 1:**
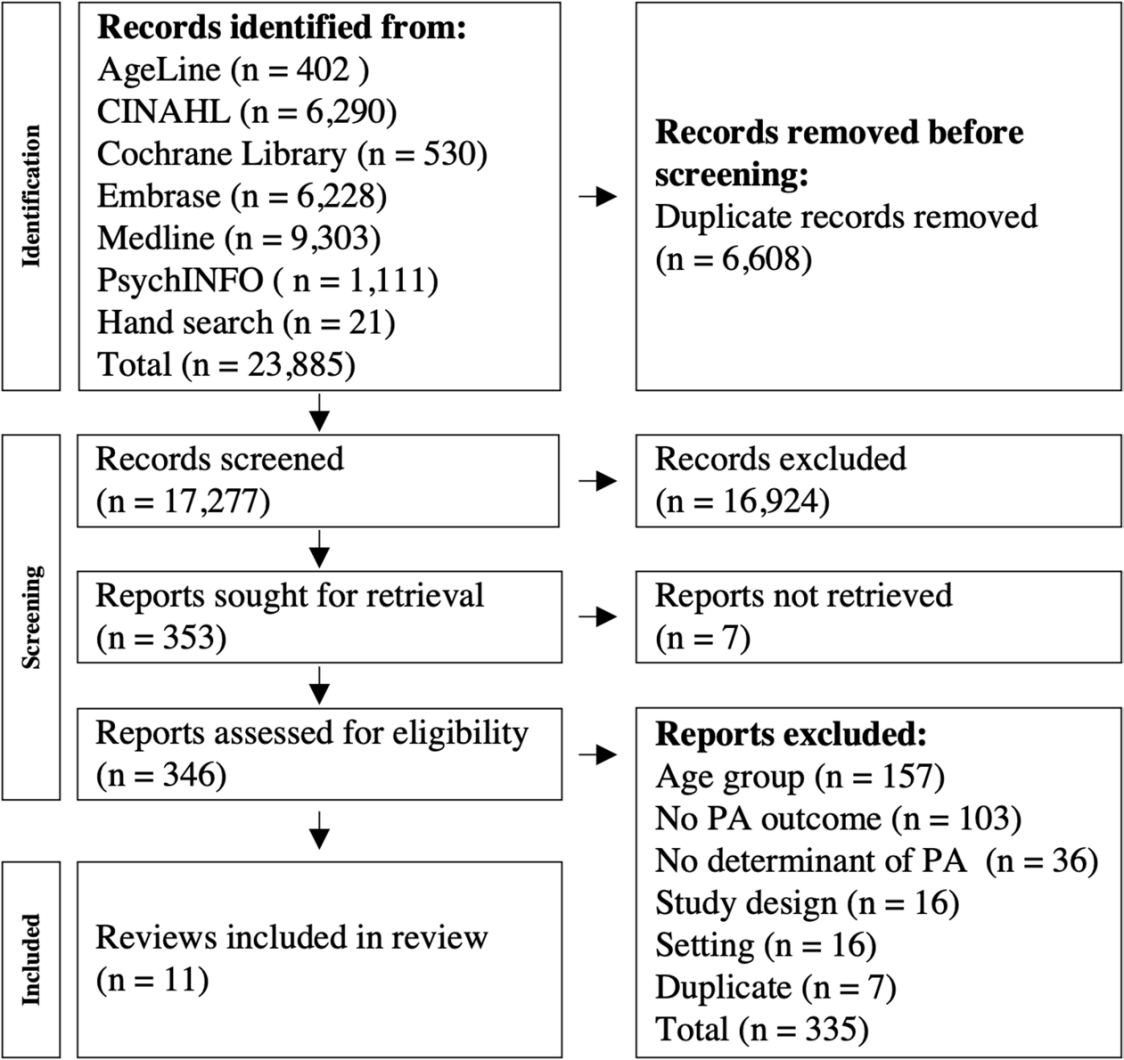
PRISMA Flow Diagram. All six databases were searched in either April or June of 2020 (20,406). An updated search was completed in Medline (Ovid) August 2022 (an additional 3,458 references found). References from included reviews were hand searched August 2022

The search results found 23,884 citations after duplicate removal, of which 346 full texts (all in English) were assessed for eligibility. Eleven reviews were included containing 410 primary studies examining PA and its determinants (Table 2 for review details). Reviews were published between 2008 [35] and 2022 [36]. Combined, the reviews searched 24 databases, with the most common being CINAHL(*n* = 9), PubMed (*n* = 8), and Medline, PsycINFO, and Web of Science (*n* = 6). Nine reviews explicitly looked at older adults, and two had populations that incidentally met our age inclusion [37, 38]. Three reviews focused on specific clinical populations living in the community, people diagnosed with dementia [38], stroke survivors [37], and individuals with subjective cognitive impairment [39]. In addition, two reviews examined determinants for more than just PA, but results for PA were synthesized separately [39, 40]. Six reviews conducted a pooled analysis in their results [34–37, 41, 42]. Eight of the eleven reviews reported the country of origin for each primary papers; 36 different countries were reported,78% from high income, 21% from upper-middle income, 1% from lower-middle income, and none of the reviews reported studies from lower-income countries (July 2021 World Bank classification) [43] (Fig. 1).

**Table 2:** Summary of review characteristics for community-dwelling older adults

Review	**Population** Age cut off (Mean age range) Sex Context	**Studies** Number (Sample size range) publication years	**Physical Activity** Type PA defined Yes/No Outcome measure	**Determinants** SEM level(s) Number included
Barnett – 2017 [42]	65 + (65–84) Mixed	*N* = 100 (44–69,253) 2001–2016	Total PA, Total MVPA, Total walking Def: No Direct and self-report	COM 7
Cerin – 2017 [34]	65 + (65–77) Mixed	*N* = 42 (44–48,879) 2004–2016	Active travel (Cycle, General, Walking) Def: Yes Self-report	COM 8
Hong – 2008 [35]	65 + ^a^ (68.4 (SD5.7)) Mixed	*N* = 37 (Total 3,389) NR	An exercise program Def: No Adherence	INTRA, ORG 9
Lindsay Smith – 2017 [44]	60 + (66–83) Mixed	*N* = 27 (64–14,072) 1992–2014	Combined, Leisure, Transport PA Def: No Self-report	INTER 4
Rosso – 2011[40]	60 + (69.8–78.5) NR	*N* = 17^b^ (105–937,857) 2004–2010	Walking Def: No Direct and Self-report	COM 7
Sun – 2013 [45]	60 + (65.8–76.1) Mixed	*N* = 53 (54–43,259) 2000–2010	Meeting PA guidelines Def: No Direct and self-report	INTRA, COM 3
Van Cauwenberg – 2018 [41]	65 + (65–79) Mixed	*N* = 72 (44–69,253) 2000–2017	Leisure-time PA (cycling, walking, overall) Def: Yes Self-report	COM 8
Yau – 2022 [36]	60 + (66–83) Mixed	*N* = 17 (11–148) 1997–2019	Exercise program Def: No Adherence	ORG 11
**Specific clinical populations in community-dwelling settings**				
Stubbs – 2014 [38]	NR (73–85.6) Mixed Dementia	*N* = 12 (24–156) 1998–2013	General PA Def: No Direct and self-report	INTRA, INTER 28
Thilarajah – 2018 [37]	NR (54–76) Mixed Stroke survivors	*N* = 26 (10–321) 2005–2016	General PA, Walking Def: Yes Direct and self-report	INTRA, INTER 20
Wion – 2019 [39]	60 + (62.8–74.67) Mixed Subjective cognitive impairment	*N* = 9^b^ (54–38,777) 2009–2018	Exercise, MVPA, General PA Def: Yes Self-report	INTRA 1

*Abbreviations*: *PA* physical activity, *SEM* Socioecological model, *SD* standard deviation, *Def* definition, *NR* not reported in review, *MVPA* moderate to vigorous physical activity, *COM* community, *INTRA* intrapersonal, *INTER* interpersonal, *ORG* organizational

^a^Used as a key term in search strategy not inclusions/exclusions

^b^Only a subgroup of these were related to the relationship between determinants and physical activity as the dependent variable

### Characteristics of primary papers in the included reviews

Over 300 unique primary papers are included in this umbrella review (Table 3). Eight reviews included at least one longitudinal study; however, two only included cross-sectional analyses of the longitudinal studies [38, 39]. Overall, 80% of the studies from included reviews were cross-sectional, 6% longitudinal, and 14% experimental. Two reviews did not report on sex of primary papers [35, 39], with 22% of all primary papers not reporting proportions of females and males. Of those reporting sex, 70% reported mixed male and female populations, 8% were female only, and less than one percent reported males only. Four reviews reported on the use of covariates in the analysis of the primary papers [34, 41, 42, 44]. From these reviews, the most common covariates included in the analyses of PA determinants were age (77%), sex/gender (61%), education (60%), a measure of health status (31%), physical function (28%), income (25%), and ethnicity/culture (25%).

**Table 3:** Characteristics of primary papers included in the reviews

Review	Number of studies included from the review	Percent of studies included in review			
		**Study designs** (XS/Long/Exp/NR)	**Sex inclusion** (F/M/Mixed/NR)	**Reported use of covariates in analysis** (Yes/No/NR)	**Type of PA outcomes** (Direct/SR/Both/Other/NR)
Barnett – 2017 [42]	100	XS 94%, Long 5%, Exp 1%	F 11%, M 2%, Mixed 81%, NR 4%	Y 82%, N 18%	D 20%, SR 73%, Both 5%
Cerin – 2017 [34]	42	XS 100%	F 2% Mixed 93% NR 5%	Y 93%, N 7%	SR 86%, NR 14%
Hong – 2008 [35]	37^b^	NR100%	NR100%	Y-MA	Adherence (100%)
Lindsay Smith – 2017 [44]	27	XS 81%, Long 11%, Exp 8%	F 15%, Mixed 81%, NR 4%	Y 67%, N 33%	D 11%, SR 85%, Both 4%
Rosso – 2011[40]	14^a^	XS 79%, Long 21%	F 8%, NR 92%	NR 100%	SR 100%
Sun – 2013 [45]	53^b^	XS 92%, Long 8%	F 11%, Mixed 42%, NR 47%	NR 100%	D 8%, SR 89%, Both 3%
Van Cauwenberg – 2018 [41]	71	XS 99%, Long 1%	F 7%, M 1%, Mixed 90%, NR1%	Y 92%, N 8%	SR 80%, NR 20%
Yau – 2022 [36]	17^b^	Exp 100%	F 18%, Mixed 82%	Y-MA	Adherence (100%)
**Specific clinical populations in community-dwelling settings**					
Stubbs – 2014 [38]	12	XS 58%, Long^c^ 33%, Exp 9%,	Mixed 92%, NR 8%	NR 100%	D 33%, SR 67%,
Thilarajah – 2018 [37]	26	XS 92%, Long 8%	Mixed 96%, NR 4%	NR 100%	D 65%, SR 19% Both 16%
Wion – 2019 [39]	4^a^	XS 75%, Long^c^ 25%	NR 100%	NR 100%	SR 100%

*Abbreviations*: *XS* cross-sectional, *Long* longitudinal, *Exp* experimental, *NR* not reported, *F* females, *M* males, *Y* yes, *N* no, *D* direct (e.g., accelerometer), *SR* self-report (e.g., questionnaire), *Both* Review included both a direct measure and self-report measure of PA, *MA* adjusted meta-analysis was performed in review

^a^Only a subgroup of the studies from a review were summarized, characteristics are reported for the subgroup only

^b^Statistics for subgroup of studies extracted not available – whole review reported

^c^Measure of PA was cross sectional in all analyses

### Characteristics of physical activity outcomes

Only three reviews cited a definition of PA [37, 39, 41], and one review provided a description specific to their review (Table S6) [34]. General or total PA was assessed in six reviews [37–39, 42, 44, 45]. Most reviews examined multiple types of PA, five reviews examined some form of walking or transportation (walking or cycling) [34, 40–42, 44], and two reviews focused on exercise programs [35, 36]. Other types of PA included in the reviews were: moderate to vigorous PA [39, 42, 45], leisure time PA [41, 44], and exercise [35, 36, 39].

There was considerable variation in how PA was captured across primary papers and reviews. Self-report measures were the most common, reported by 79% of primary papers; popular measures were the Community Healthy Activities Model Program for Seniors (*n* = 35), International Physical Activity Questionnaire (*n* = 34), Physical Activity Scale for the Elderly (*n* = 16), and the Physical Activity Questionnaire (*n* = 14). Eighteen percent of papers used a direct measure of PA (15% used only direct measures); 30 studies used an accelerometer, 12 studies used a pedometer, and one included global positioning systems (GPS). Only 3% of primary papers used both a self-report and direct measure. Of all included primary papers 6% did not report the PA outcome measure used. Even greater variation was seen for PA reporting when operationalizing the outcomes used from the measures (e.g., dichotomous, categorical and continuous outcomes). See Table S7 which demonstrates this point, summarizing over 50 reported outcomes just related to walking.

### Determinants of physical activity

Determinants were mapped using the SEM to the community, intrapersonal, and interpersonal levels. The most common levels examined were intrapersonal determinants [35, 37–39, 45] and the community [34, 40–42, 45]; however, all the community level determinants were from the physical environment. There was variability in the naming of determinants by the review authors resulting in large numbers of unique determinants (e.g., access to/availability of services/destinations had 50 unique terms, six of which were related to parks). The physical environment had the greatest number of determinants, grouped according to the NEWS categories: access to/availability of services/destinations – general (*n* = 149), safety and traffic (*n* = 111), aesthetics & cleanliness/order (*n* = 85), pedestrian/cycling infrastructure & streetscape (*n* = 82), residential density/urbanisation (*n* = 68), street connectivity (*n* = 62), and walkability (*n* = 40). There were 27 intrapersonal determinants summarized, plus an additional eight considered specific to a clinical population, and three interpersonal determinants (full list available in Table S4).

#### Relationships reported in general community-dwelling populations

Relationships between PA and determinants were summarized for general PA (i.e., all types of PA) and adherence to PA. Across the four reviews examining physical environment determinants and general PA, the summary directions were null for five determinants, inconsistent for one, and positive for one [34, 40–42]. Where possible, direct and self-report measured PA relationships are presented separately (Table 4). Only the relationship between walkability and PA demonstrated a difference between these two types of measurements in the review by Barnett et al. (i.e., self-report IC and direct + Cor) [42]. Only two reviews included intrapersonal/interpersonal determinants. One review found a positive relationship between PA and social support specifically for PA, and a negative relationship between loneliness and PA [44]. The second review reported negative relationships between both age and female gender and PA; however, individual study relationships were not provided- only a narrative summary [45]. In the two reviews examining determinants of exercise program adherence, both found the program duration to be negatively associated in adjusted models [35, 36]. The review by Hong et al. also found the program format (i.e., groups vs individual) to be significantly related to adherence, and Yau et al. found that whether the program was supervised also contributed to adherence (Table S8).

**Table 4:** Overall direction, percent, and number of relationships between determinants and physical activity in general community-dwelling older adults

Determinant (CCA%)	Barnett 2017^a^ [42]	Barnett 2017^b^ [42]	Cerin 2017 [34]	Lindsay Smith 2017^a^ [44]	Lindsay Smith 2017^b^ [44]	Rosso 2011 [40]	Rosso 2011^b^ [40]	Sun 2013^c^ [45]	Van Cauwenberg 2018 [41]	**Summary**
*Direction (Null,* + *Cor, -Cor, Null Lim,* + *Lim, -Lim, IC) and (percent favouring overall direction and number of relationships reported)*										
**Community (COM)**										
Access to / availability of services / destinations (12.5%)	Null (77% of 242)	Null (88% of 174)	IC (128)	-	-	Null (60% of 10)	Null (100% of 4)	-	Null (73% of 191)	**Null**
Aesthetics & cleanliness / order (14.7%)	Null (77% of 45)	Null (88% of 17)	Null (74% of 42)	-	-	+ Lim (100% of 2)	-	-	Null (72% of 84)	**Null**
Pedestrian / cycling infrastructure & streetscape (15.2%)	Null (76% of 63)	Null (61% of 23)	IC (80)	-	-	Null (83% of 6)	+ Lim (100% of 1)	-	Null (77% of 97)	**Null**
Pedestrian / cycling infrastructure & streetscape – reverse relationships (29.4%)	-	-	Null (75% of 20)	-	-	-	-	-	Null (87% of 21)	**Null**
Residential density / urbanisation (13.2%)	IC (48)	Null (90% of 10)	IC (20)	-	-	+ Lim (67% of 3)	-	IC Lim (2)	Null (84% of 46)	**IC**
Safety & traffic (11.8%)	Null (71% of 102)	Null (81% of 28)	Null (65% of 80)	-	-	+ Lim (100% of 2)	-	-	Null (81% of 118)	**Null**
**Community (COM)**										
Street connectivity (21.1%)	Null (67% of 24)	Null (74% of 12)	Null (67% of 21)	-	-	IC (4)	-	-	Null (90% of 29)	**Null**
Walkability (13.8%)	IC (13)	+ Cor (75% of 8)	+ Cor (83% of 12)	-	-	+ Lim (100% of 3)	-	-	Null (69% of 15)	**Pos**
**Intrapersonal (INTRA)**										
Age	-	-	-	-	-	-	-	-Cor (NR of 18)	-	**Neg**^**d**^
Gender (female)	-	-	-	-	-	-	-	-Cor (NR of 22)	-	**Neg**^**d**^
Loneliness	-	-	-	Null Lim (1)	-Cor (70% of 5)	-	-	-	-	**Neg**^**d**^
**Interpersonal (INTER)**										
General social support	-	-	-	IC (4)	-	-	-	-	-	**IC**^**d**^
Social isolation	-	-	-	-Lim (1)	-	-	-	-	-	**Neg**^**d**^
Social support for physical activity	-	-	-	+ Cor (60% 0f 20)	+ Cor (75% of 4)	-	-	-	-	**Pos**^**d**^

Where possible, results are presented separately for self-report vs direct measures of PA (e.g., Barnett 2017a vs Barnett 2017b). Based on the cut-offs described above, an overall direction between PA and the determinant is shown for each review. The brackets contain the percent of relationships that favoured the overall direction and the number of relationships examined for each determinant. A summary is provided for each determinant representing the overall direction from the majority (i.e., > 50%) of reviews. Summaries only included the direct measure overall relationship when reviews presented both self-report and direct PA relationships. If no majority was found across reviews, the determinant was summarized as inconsistent

Corrected covered area: N—the sum of the number of primary papers in each review; r – total number of primary papers included (unique); c – number of reviews

$$\mathrm{CCA}=\frac{N-r}{rc-r}$$

*Abbreviations*: *CCA* corrected covered area, Null > 60% of relationships were not significantly related, +Cor >60% of relationships were positively related, -Cor > 60% of relationships were negatively related, *IC* inconsistent results, Lim less than four relationships were presented (limited evidence), *Neg* negative, *Pos* positive

^a^Including results from only self-reported PA

^b^Including results from only direct measure PA

^c^Narrative summary (individual study relationships not reported)

^d^Summary is based on a single review

#### Relationships reported in specific clinical populations of community-dwelling older adults

We found limited results for the majority of relationships examined (i.e., < 4 relationships summarized). For older adults diagnosed with dementia, null relationships were found between PA and age, body composition, and cognition; however, a positive relationship was found for quality of life. Wion et al. also showed that subjective cognitive impairment was negatively related to PA (Table 5). In the meta-analysis presented by Thilarajah et al. for stroke survivors, there were several significant pooled effects: age, balance, cardiorespiratory fitness, and mobility; all included at least four studies (Table 6 summary of pooled analyses).

**Table 5:** Overall direction, percent, and number of relationships between determinants and physical activity in community-dwelling clinical populations

Determinant	Stubbs 2014^a^ [38] **Dementia**	Stubbs 2014^b^ [38] **Dementia**	Thilarajah 2021^a^ [37] **Stroke survivors**	Thilarajah 2021^b^ [37] **stroke survivors**	Wion 2019 [39] **Subjective cognitive impairment**
*Direction (Null,* + *Cor, -Cor, Null Lim,* + *Lim, -Lim, IC) and (percent favouring overall direction and number of relationships reported)*					
** Intrapersonal (INTRA)**					
Age	Null (100% of 5)	-Lim (67% of 3)	-	-	-
Balance	Null Lim (1)	-	-	-	-
Body composition	Null (75% of 4)	Null (60% of 5)	-	-	-
Cardiorespiratory fitness	Null Lim (1)	-	-	+ Lim (100% of 2)	-
Cognition	Null (86% of 7)	Null Lim (100% of 3)	IC (2)	-	-
Depression	IC (2)	Null Lim (100% of 2)	-Cor (4)	IC (2)	-
Dizziness	-Lim (1)	-	-	-	-
Education	+ Lim (100% of 2)	Null Lim (100% of 2)	-	-	-
Energy intake	-	+ Lim (1)	-	-	-
Ethnicity	-	Null Lim (1)	-	-	-
Gender	-	Null Lim (1)	-	-	-
History of falls	-Lim (1)	-	-	-	-
Mobility	IC (6)	-Lim (1)	-Lim (1)	+ Lim (100% of 2)	-
Number of comorbidities	Null Lim (1)	-	-	-	-
Number of medications	-Lim(1)	-	-	-	-
Physical function	Null Lim (100% of 2)	+ Lim (1)	-	IC (2)	-
Quality of life	+ Cor (80% of 5)	-	IC (2)	-	-
Resting metabolic rate	-	+ Lim (1)	-	-	-
Self-efficacy - balance	-	-	-	+ Lim (100% of 2)	-
Self-efficacy - exercise	-	-	+ Lim (1)	-	-
Self-efficacy - general	-	-	-	Null Lim (1)	-
Sleep	IC (2)	Null Lim (1)	-	+ Lim (1)	-
Social functioning	+ Lim (1)	-	-	-	-
Socioeconomic status	Null Lim (1)	-	-	-	-
Subjective cognitive impairment	-	-	-	-	-Cor (75% of 4)
Waking hours in a day	-Lim (1)	-	-	-	-
**Disease related determinants**					
Apathy	+ Lim (1)	-	-	-	-
Autonomic function	-Lim (1)	-	-	-	-
Delirium	-Lim (1)	-	-	-	-
Dementia - behavioural function	Null (5)	-	-	-	-
Dementia - duration	Null Lim (67% of 3)	-	-	-	-
Dementia - severity	Null Lim (1)	-	-	-	-
Left side infarct	-	-	-	-Lim (1)	-
Quality of life - stroke	-	-	+ Lim (1)	-	-

Where possible, results are presented separately for self-report vs direct measures of PA (e.g., Stubbs 2014a vs Stubbs2014b). Based on the cut-offs described above, an overall direction between PA and the determinant is shown for each review. The brackets contain the percent of relationships that favoured the overall direction and the number of relationships examined for each determinant. A summary was not created due to heterogeneity of populations

*Abbreviations*: *Null* > 60% of relationships were not significantly related, + *Cor* > 60% of relationships were positively related, *-Cor* > 60% of relationships were negatively related, *IC* inconsistent results (< 60% of results favour all directions), *Lim* limited—less than 4 relationships were presented

^a^Including results from only self-reported PA

^b^Including results from only direct measure PA

**Table 6:** Direction and number of pooled effects for determinants and physical activity

Determinant	Barnett 2017 [42]	Cerin 2017 [34]	Van Cauwenberg 2018 [41]	Thilarajah 2021 [37] **Stroke survivors**	**Summary**
*Direction of relationship (Null,* + *ES, -ES) and (number of corresponding pooled ES)*					
**Community (COM)**					
Access to / availability of services / destinations	Null (34) + ES (16)	Null (26) + ES (21)	Null (47) + Cor (7)	-	**Null**
Aesthetics & cleanliness / order	Null (3) + ES(3)	Null (13) -ES (1)	Null (14) + ES (2)	-	**Null**
Pedestrian / cycling infrastructure & streetscape	Null (10) + ES (4)	Null (5) + ES (12)	Null (28) + ES (1)	-	**Null**
Pedestrian / cycling infrastructure & streetscape – reverse relationships	-	Null (4)	Null (2) -ES (1)	-	**Null**
Residential density / urbanisation	Null (3) + ES (1)	Null (2) + ES (2) -ES(1)	Null (5)	-	**Null**
Safety & traffic	Null (5) + ES (3)	Null (10) + ES (2)	Null (16) + ES (3) -ES (1)	-	**Null**
Street connectivity	Null (4)	Null (1) + ES (3)	Null (5)	-	**Null**
Walkability	+ ES (4)	Null (1) + ES(3)	Null (4) + ES(1)	-	**Pos**
**Intrapersonal (INTRA)**					
Age	-	-	-	-ES (1)	**Neg**^c^
Balance	-	-	-	+ ES (1)	**Pos**^c^
Cardiorespiratory fitness	-	-	-	+ ES (2)	**Pos**^c^
Cognition	-	-	-	Null^b^ (1)	**Null**^c^
Fatigue	-	-	-	-ES (1)	**Neg**^c^
Impairment	-	-	-	+ ES (1)	**Pos**^c^
Mobility	-	-	-	+ ES^b^ (1)	**Pos**^c^
Physical function	-	-	-	+ ES^b^ (1)	**Pos**^c^
Self-efficacy - falls	-	-	-	-ES^a^ (1)	**Neg**^c^
Sex	-	-	-	-ES (1)	**Neg**^c^
Stroke impact scale	-	-	-	-ES (1)	**Neg**^c^
Years since stroke	-	-	-	Null^b^ (1)	**Null**^c^

For pooled analyses, relationships were summarized by providing the number of effect estimates favouring each relationship direction. The summary for each determinant is the direction in which the majority (i.e., 50%) of reviews favoured. A review was said to favour a direction if 60% or more of the pooled effects were the same. For example, Barnett 2017 had 34 null effect estimates and 16 positive effect estimates between PA and access to/availability of services/destinations. Therefore, > 60% favoured null for both Barnett 2017 [42] and Van Cauwenberg 2018 [41], meaning the summary (i.e., majority of reviews) was null. The summary excludes clinical populations living in the community

*Abbreviations*:* Null* relationships were not significant, + *ES* effect estimates were positive, *-ES* effect estimates were negative, *IC* inconsistent results

^a^including results from only self-reported physical activity

^b^including results from only direct measure physical activity

^c^summary based on a single review

### Assessment of methodological quality and quality of the evidence

Of the eleven criteria assessed on the JBI critical appraisal checklist four were consistently met, appropriate inclusion criteria (100%), clear/explicit research question (100%), directives for new research (91%) and appropriate recommendations for policy (80%). The areas of greatest concern were the sources and resources used in searches and the search strategies, specifically the justifications around restrictions (e.g., only English). All criteria are summarized in Figure S1. Sixty-four percent of the reviews performed some form of risk of bias or quality assessment of the included primary papers; however, none of the included reviews assessed the overall quality of the evidence for different outcomes across primary papers.

### Corrected cover area

The overall CCA for this umbrella review was slight (2.5%). However, Hong et al. was excluded from the calculation as we were unable to determine the primary papers included in the review (*n* = 37). Calculations were completed for determinants examined by multiple reviews. The CCAs for the physical environment determinants ranged from 11.8% (high) to 29.4% (very high indicating overlap between primary papers included by reviews examining these determinants.

## Discussion

This is the first umbrella review to synthesize the evidence on determinants of PA in community-dwelling older adults. Although our review included > 300 unique primary papers, with a CCA of just 2.5%, the majority of studies examined only the physical environment and much of the existing research failed to demonstrate consistent relationships. Nonetheless, we did identify several determinants significantly related to PA in general populations of community-dwelling older adults: social support for PA, loneliness, age, gender, and walkability. In addition, we noted the following knowledge gaps for future studies to address: 1) less than half of the reviews included a definition of PA, 2) over 75% of relationships examined used a self-reported measure of PA and, 3) only 6% of relationships summarized used a longitudinal study design.

Our umbrella review found evidence for several determinants of PA in community-dwelling older adults. Based on the SEM, the physical environment was the predominant type of determinant examined within the community level influences on PA and as compared to intrapersonal or interpersonal factors. Of the seven physical environmental determinants examined, only walkability had results consistent enough to suggest a relationship with PA (higher walkability score with greater PA), including pooled effects that accounted for sample size and quality of primary papers [34, 42]. This is consistent with results of an umbrella review on physical environment determinants in adults 18 + [15]. Unlike the other physical environment determinants, walkability is an index, made up of street connectivity, residential density, and land-use mix, rather than a single construct such as access to a specific building or service [34, 41]. Therefore, a possible explanation could be that individual physical environment determinants alone may not have enough of an effect but rather an accumulation of effects is needed to influence PA behaviour.

Most of the relationships examined outside of the physical environment had either limited evidence (relationships assessed < 4 times) or were only reported in a single review. The paucity of evidence for determinants outside the physical environment is not exclusive to older adults and has been reported previously for general adult populations [46]. Despite only being reported by a single review, three determinants still had a considerable number of studies supporting their relationships with PA. In Lindsay et al., a positive relationship between social support for PA and PA was supported by 15 of 24 studies. While Sun et al. did not provide individual study evidence for the negative relationships between age and PA or gender and PA, they reported that 18 and 22 studies examined each, respectively. Given the overall limited research on interpersonal determinants (i.e., social environment) in community-dwelling older adults and the strong evidence for the relationship between social support of PA and PA, we believe further study of social (i.e., interpersonal) determinants is warranted.

The heterogeneity in how PA was defined and measured is another notable finding of our umbrella review; a point which has been acknowledged in previous work [15]. Only four of the eleven included reviews defined PA. In many cases, authors would provide a general term or report the way they categorized/grouped PA (e.g., leisure time PA, MVPA) but did not further define what would fall under each category. These categories tended to overlap (e.g., leisure-time PA, active travel, MVPA) making it harder to distinguish between different types of PA. By grouping PA into a single category, we worked around the ambiguity of PA types included in reviews; however, the lack of standardization may be confounding the results if determinants vary by the type of PA. The confusion regarding what PA is being captured is compounded by the number of PA outcome measures used (*n* = 50, Table 7), since not all PA measures capture the same diversity of activities. For example, some questionnaires more comprehensively capture light-intensity activities compared to others [47, 48]. Overall, measurement of PA was a strong source of heterogeneity in the relationships summarized in this umbrella review. To improve our understanding of determinants of PA it is important that future research clearly defines what PA is being examined a priori, and where appropriate, provide a description of the types of PA that were captured by the measures used.

**Table 7:** Types of physical activity measures (number of different tools/instruments listed)

**Standardised Self-reported Questionnaires** (*n* = 24)
Active Australia survey, Baecke Physical Activity Questionnaire, Community Healthy Activities Model Program for Seniors (CHAMPS), Cross-cultural activity questionnaire, EPIC Physical activity questionnaire, Global Physical Activity questionnaire (GPAQ), Godin-Shephard Leisure-Time Physical Activity Questionnaire, Human activity profile, Incidental and Planned Exercise questionnaire, International Physical Activity Questionnaire (IPAQ), international social survey programme, Leisure time physical activity questionnaire, Neighbourhood Physical Activity questionnaire, Neighbourhood Walkability questionnaire—modified for Chinese seniors (NWQ), Nordic Physical activity Questionnaire, Paffenbarger Activity questionnaire, Physical activity questionnaire, Physical activity questionnaire for older Thai persons, Physical Activity Scale for individuals with Physical Disabilities, Physical Activity Scale for the Elderly (PASE), Short Questionnaire to Assess physical activity, World Health Organisation STEPS questionnaire, Yale Physical Activity Scale questionnaire (YPAS)
**Reported items or scales from cohort studies** (*n* = 19)
**Accelerometers** (*n* = 3)
**Pedometers** (*n* = 1)
**Diary/logs**
**Single question/frequency/quantity**
**Unnamed survey/questionnaire**
**Not reported**

In addition to the sheer number of measures used to capture PA, there was a predominance of self-report measures compared to direct measures of actual PA in daily life such as step count or activity intensity via wearable devices. Only 18% of primary papers included a direct measure, compared to the 79% that included a self-report measure. Self-report measures of PA may increase the indirectness of measurement, decreasing the quality of evidence [49], as suggested by the low convergent validity values between direct measures of PA like step count or double labeled water (e.g., *r* = 0.3–0.59) [48, 50, 51]. However, questionnaires or tools like logs/diaries still provide much needed information on the context (e.g., the kinds of activity completed) in which the activity is done; information which is missing from some direct measurement approaches (e.g., pedometer). In recent years, the accessibility (e.g., cost, product availability) and feasibility (e.g., usability, burden) of activity monitoring devices has greatly improved increasing their acceptability for use in older populations [52–54]. Moving forward, researchers should consider using such approaches alongside self-report measures to support our understanding of PA behaviour in older adults.

Of the greater than 300 primary papers included in our umbrella review, only 6% used a longitudinal design. Cross-sectional study designs are not able to identify predictors of future PA behaviours or trajectories. This is a gap in the current evidence on PA in older adults that also applies to other populations [14–16]. Longitudinal relationships provide critical information for examining change in PA behaviour over time in the presence of different potential determinants. This information could be used to identify subgroups of people who may be at greater risk for decreasing PA levels as well as to identify determinants that can be promoted through policy to improve PA levels.

### Limitations

There are several limitations of this umbrella review. First, our team chose to restrict the setting to community-dwelling older adults; during full text screening reviewers noted that in many cases the setting was either poorly defined/reported or mixed. This may have resulted in the exclusion of potentially relevant evidence. However, due to the high CCA rating for physical environment determinants, we have relatively high confidence that we captured a representative sample of the current state of the evidence for these determinants. The use of the CCA is a recommended measure of overlap between reviews in umbrella reviews [30], but it does not accurately capture some of nuances in this overlap. For example, if a primary paper presented multiple PA outcomes, it does not account for whether both reviews extracted the same relationships, which would result in the CCA overestimating the overlap. Therefore, while CCA is useful to understand the presence of overlap, ratings should be interpreted with caution. Finally, while this umbrella review aimed to study determinants in the broadest sense of the term, most summarized studies were cross-sectional; therefore, many of the examined determinants would be more appropriately termed correlates. Despite these limitations this umbrella review still provides an overview of the current literature (> 300 primary papers) using strong methodological rigour, following the JBI guidelines and a published protocol, with the assistance of a health research librarian (NB).

## Conclusion

This umbrella review found evidence supporting walkability, age, gender, loneliness, and social support for PA as determinants of PA in older people living in the community. These findings should be interpreted with caution given the heterogeneity of PA outcomes, potential indirectness of the measures used, and the dearth of longitudinal studies. Therefore, despite the large quantity of research conducted on PA to date, the results of this umbrella review support the need for continued research on relationships between determinants and PA in older adults, especially longitudinal studies and that focus on influences beyond the physical environment. Future research on PA in older adults should include explicit definitions of PA and the types of PA (e.g., leisure-time, total PA, MVPA) under investigation, as well as standardization around outcome operationalization. We also encourage the use of direct measures of PA such as those obtained via wearable activity monitors to improve our understanding of relationships between determinants and trajectories of PA over time.

### Supplementary Information

**Additional file 1:  Table S1.** Ovid Medline 1946 to August 1^st^, 2022. **Table S2.** Ratings for Assessment of Methodological Quality. **Table S3.** Community determinant categorization. **Table S4.** Interpersonal determinant categorization. **Table S5.** Intrapersonal determinant categorization. **Table S6.** Physical activity definitions and inclusion. **Table S7.** Examples of reported physical activity outcomes with respect to walking. **Table S8.** Direction and pooled effects from meta-analytical results of relationships between determinants and exercise program adherence. **Figure S1.** Summary of JBI Critical Appraisal Checklist for the eleven included reviews. **Box S1.** Reported Items or scales from Cohort studies (*n*=19). **Table S9.** Full-text Exclusion Reason.

## Data Availability

All data extracted and analysed during this study are available upon request from the corresponding author.

## Abbreviations

AMSTAR2: Assessing the Methodological Quality of Systematic Reviews version 2
CCA: Corrected covered area
Cor: Correlated (> 60% of relationships favoured that direction)
GRADE: Grading of Recommendations, Assessment, Development and Evaluation
IC: Inconsistent
Lim: Limited evidence (relationship examined < 4 times)
JBI: Joanna Briggs Institute
MVPA: Moderate to vigorous physical activity
NEWS: Neighborhood Environment Walkability scale
NR: Not reported
PA: Physical activity
PROSPERO: International prospective register of systematic reviews
SEM: Socioecological model
WHO: World Health Organization
