# Supplementary material for: Determinants of physical activity in community-dwelling older adults: an umbrella review
Source: Int J Behav Nutr Phys Act. 2023 Nov 21;20:135. doi: 10.1186/s12966-023-01528-9 (PMC10664504; doi:10.1186/s12966-023-01528-9)
Supplement: Supplementary file 1 — Additional file 1: Table S1. Ovid Medline 1946 to August 1st, 2022. Table S2. Ratings for Assessment of Methodological Quality. Table S3. Community determinant categorization. Table S4. Interpersonal determinant categorization. Table S5. Intrapersonal determinant categorization. Table S6. Physical activity definitions and inclusion. Table S7. Examples of reported physical activity outcomes with respect to walking. Table S8. Direction and pooled effects from meta-analytical results of relationships between determinants and exercise program adherence. Figure S1. Summary of JBI Critical Appraisal Checklist for the eleven included reviews. Box S1. Reported Items or scales from Cohort studies (n=19). Table S9. Full-text Exclusion Reason. [file 12966_2023_1528_MOESM1_ESM.docx]

Determinants of physical activity in community-dwelling older adults: an umbrella review – Supplementary Appendices

Table of Contents

[Search Strategy 2](#_Toc140218611)

[Table S1. Ovid Medline 1946 to August 1^st^, 2022 2](#_Toc140218612)

[Assessment of Methodological quality details 5](#_Toc140218613)

[Table S2. Ratings for Assessment of Methodological Quality 5](#_Toc140218614)

[Determinant Category Grouping 6](#_Toc140218615)

[Table S3. Community determinant categorization 6](#_Toc140218616)

[Table S4. Interpersonal determinant categorization 8](#_Toc140218617)

[Table S5. Intrapersonal determinant categorization 8](#_Toc140218618)

[Physical activity definitions and inclusion summary 9](#_Toc140218619)

[Table S6. Physical activity definitions and inclusion 9](#_Toc140218620)

[Physical activity outcome operationalization example 12](#_Toc140218621)

[Table S7. Examples of reported physical activity outcomes with respect to walking 12](#_Toc140218622)

[Relationship summary between determinants and adherence to exercise 12](#_Toc140218623)

[Table S8. Direction and pooled effects from meta-analytical results of relationships between determinants and exercise program adherence 12](#_Toc140218624)

[Summary of JBI Critical Appraisal Checklist 13](#_Toc140218625)

[Figure S1. Summary of JBI Critical Appraisal Checklist for the eleven included reviews 14](#_Toc140218626)

[Additional self-report instrument summary 14](#_Toc140218627)

[Box S1. Reported Items or scales from Cohort studies (n=19) 14](#_Toc140218628)

[Socioecological model 15](#_Toc140218629)

[Full-text Exclusion Reasons 16](#_Toc140218630)

[Table S9. Full-text Exclusion Reason 16](#_Toc140218631)

# Search Strategy

## Table S1. Ovid Medline 1946 to August 1^st^, 2022

| 1 | exp Exercise/ |
| --- | --- |
| 2 | exp Physical Fitness/ |
| 3 | exp sports/ |
| 4 | exp Recreation/ |
| 5 | running/ or swimming/ or walking/ |
| 6 | Motor Activity/ |
| 7 | (physical$ adj5 (fit$ or physical$ or activ$)).tw,kf. |
| 8 | (exercis$ adj5 (train$ or physical$ or activ$)).tw,kf. |
| 9 | (sport$ or walk$ or bicycle$).tw,kf. |
| 10 | (exercise$ adj aerobic$).tw,kf. |
| 11 | ((lifestyle or life style) adj5 activ$).tw,kf. |
| 12 | Yoga/ |
| 13 | exp yoga/ or exp fitness centers/ |
| 14 | (fitness adj (regime* or program*)).ti,ab,kf. |
| 15 | (cardiorespiratory fitness or aerobic capacity or (led walk* or health walk*)).ti,ab,kf. |
| 16 | ((moderate or vigorous* or low) adj activ*).ti,ab,kf. |
| 17 | ((exercis* or physical) adj5 (fit* or train* or activ* or endur*)).ti,ab,kf. |
| 18 | ((leisure or fitness) adj5 (centre* or center* or facilit*)).ti,ab,kf. |
| 19 | ((promot* or uptak* or encourag* or increas* or start* or adher* or sustain* or maintain*) adj5 (circuit* or aqua* or gym* or physical active*)).ti,ab,kf. |
| 20 | ((decreas* or reduc* or discourag*) adj5 (sedentary or deskbound or "physical* inactiv*" or physical activit*)).ti,ab,kf. |
| 21 | ((cycle or cycling) adj5 (travel$ or facilit$ or park$ or friendly or infrastructure)).ti,ab,kf. |
| 22 | (bicycl* or (bike*1 or biking) or (swim*1 or swimming) or (exercis*3 adj5 aerobic*) or rollerblading or rollerskating or skating or strength training or weight lift* or weightlift*).tw,kf. |
| 23 | ("use" adj3 stair*).ti,ab,kf. or recreation*1.ti,kf. |
| 24 | (exercis$ adj5 physical$).mp. |
| 25 | (leisure* adj5 activ*).mp. |
| 26 | Sedentary behavior/ |
| 27 | Sedentary.tw,kf. |
| 28 | Leisure Activities/ |
| 29 | Exercise*.tw,kf. |
| 30 | (sport*3 or walk*3 or running or jogging or pilates or yoga).ti,ab,kf. |
| 31 | ((cycle or cycling) adj5 (travel$ or facilit$ or park$ or friendly or infrastructure)).ti,ab,kf. |
| 32 | (recreation*1 or ("use" adj3 stair*)).ti,ab,kf. |
| 33 | healthy lifestyle/ or healthy aging/ |
| 34 | (active lifestyle* or activity guideline*).mp. |
| 35 | ((resistance or strength* or resilience) adj5 train*).mp. |
| 36 | or/1-35 |
| 37 | Health Behavior/ |
| 38 | Health Status/ |
| 39 | Sedentary.tw,kf. or Sedentary behavior/ |
| 40 | exp self efficacy/ |
| 41 | exp Socioeconomic Factors/ |
| 42 | leisure*.mp. |
| 43 | exp life style/ |
| 44 | Patient Dropouts/ or dropout*.mp. |
| 45 | exp Residence Characteristics/ |
| 46 | (life style* or lifestyle*).mp. |
| 47 | exp health promotion/ |
| 48 | Motivation/ or motivation*.mp. |
| 49 | (motivat* or proactivit* or predict* or retire* or determinant* or correlate* or (neighbourhood* or neighborhood*) or barrier*).mp. |
| 50 | (adher* or adherence* or underadher* or under adher*).mp. |
| 51 | Environment/ or environment*.mp. |
| 52 | (facilit* or walkabil* or bikeabil* or aesthetic* or greenness or park or parks or open space*).mp. |
| 53 | Social Support/ |
| 54 | Automobile Driving/ or driving status.mp. |
| 55 | (promot* or attribute* or behavior* or behaviour*).tw,kf. |
| 56 | (socioeconomic* or (socio ecologic* or socioecologic*) or moderator* or enable* or factor*).mp. |
| 57 | Environment Design/ |
| 58 | relationship*.tw,kf. |
| 59 | participat*.tw,kf. |
| 60 | attitude to health/ |
| 61 | exp social behavior/ |
| 62 | (uptak* or encourag* or increas* or start* or sustain* or maintain*).tw,kf. |
| 63 | (decreas* or reduc* or discourag*).tw,kf. |
| 64 | (active adj (travel*4 or transportation or commut$)).tw,kf. |
| 65 | ((cycle or cycling) adj5 (commut$ or travel$ or facilit$ or park$ or friendly or infrastructure)).ti,ab,kf. |
| 66 | recreation*1.ti,ab,kf. |
| 67 | psychology/ or exp cognitive science/ or psychology, social/ |
| 68 | mobility.mp. or Mobility Limitation/ |
| 69 | psychosocial.tw. |
| 70 | exp Cognition/ |
| 71 | cognition.tw,kf. |
| 72 | exp Executive Function/ or executive function*.mp. |
| 73 | exp social support/ |
| 74 | social network*.mp. |
| 75 | exp Social Networking/ |
| 76 | exp Biological Factors/ |
| 77 | exp Health Risk Behaviors/ |
| 78 | (socio demograph* or sociodemograph*).tw,kf. |
| 79 | or/37-78 |
| 80 | exp Aged/ |
| 81 | (retirement or retired).mp. |
| 82 | (late* life or aged).mp. |
| 83 | old age*.mp. |
| 84 | (old* adj3 (person* or people or adult* or wom#n or man or men or female* or male* or individual*)).mp. |
| 85 | frail*.ti,ab,kf. |
| 86 | (geriatr* or elder* or gerontol* or senior* or frail).mp. |
| 87 | (young old or old old or oldest old or centenarians).ti,ab,kf. |
| 88 | Aging/ or aging.mp. |
| 89 | senescence.ti,ab,kf. |
| 90 | or/80-89 |
| 91 | meta analysis.pt. |
| 92 | meta anal$.mp. |
| 93 | metaanal$.mp. |
| 94 | metanal$.mp. |
| 95 | systematic review$.mp. |
| 96 | systematic overview$.mp. |
| 97 | ((pool: or combined or combining) adj (data or trial* or studies or results)).mp. |
| 98 | ((hand adj2 search:) or handsearch:).mp. |
| 99 | cochrane.mp. |
| 100 | ((quantitative or systematic: or methodologic: or integrative:) adj2 (review: or overview: or synthes: or survey:)).mp. |
| 101 | (peto or der simonian or dersimonian).mp. |
| 102 | or/91-101 |
| 103 | (pooled analys: or pooling or mantel haenszel:).mp. |
| 104 | fixed effect:.mp. |
| 105 | (extraction or medline or embase or pubmed or cinahl).ab. |
| 106 | 103 or 104 or 105 |
| 107 | (review: or cochrane).mp. |
| 108 | 105 and 106 |
| 109 | 102 or 108 |
| 110 | animals/ not humans/ |
| 111 | 109 not 110 |
| 112 | scoping review*.mp. |
| 113 | (meta synthes* or metasynthes* or meta regression or metaregression).mp. |
| 114 | 112 or 113 |
| 115 | 114 not 110 |
| 116 | 111 or 115 |
| 117 | 36 and 79 and 90 and 116 |
| 118 | limit 117 to yr="2020 -Current" |

# Assessment of Methodological quality details

| Table S2. Ratings for Assessment of Methodological Quality | | |
| --- | --- | --- |
| JBI Item | Criteria used | Comment |
| Is the review question clearly and explicitly stated? | AMSTAR Question 1   - Included population - Included intervention - Included comparator - Included outcome | If comparator NA count as yes |
| Were the inclusion criteria appropriate for the review question? | AMSTAR Question 1 | If comparator NA count as yes |
| Was the search strategy appropriate? | AMSTAR Question 4   - incl keywords/search strategy - justified pub restrictions | Yes – requires yes to both |
| Were the sources and resources used to search for studies adequate? | AMSTAR Question 4   - 2+ databases searched - Search reference lists of included studies - Searched registries - Consulted content expert - Searched Grey literature - Search was conducted within 24months |  |
| Were the criteria for appraising appropriate? | AMSTAR Question 9   - Examined confounding - Examined selection bias - Examined methods to decide exposure - Selection of reported results |  |
| Was the critical appraisal conducted by 2 or more reviewers independently? | Not mapped to AMSTAR |  |
| Were there methods to minimize errors in data extraction? | AMSTAR Question 6   - At least 2 reviewers extracted - 1 extracted but 2 piloted and achieved >80% agreement | Yes, to one or the other |
| Were there methods used to combine studies appropriate? | AMSTAR Question 11   - Justified combining data - Adjusted for heterogeneity - Investigated heterogeneity - Separate estimates for RCTs and NRSIs |  |
| Was the likelihood of publication bias assessed? | AMSTAR Question 15   - Tested and discussed publication bias |  |
| Were the recommendations for policy and/or practice supported by the reported data? | Not mapped to AMSTAR |  |
| Were the specific directives for new research appropriate? | Not mapped to AMSTAR |  |
| Assessment of quality of the evidence | | |
| Did they assess quality of the evidence (GRADE) |  |  |

# Determinant Category Grouping

| Table S3. Community determinant categorization | |
| --- | --- |
| **Category used in umbrella review** | **Determinants (terms) from reviews included in this category** |
| Access to / availability of services / destinations | Access / availability of services other, access to destination / services, access to services / destination, business / government / institutional / industrial, commercial/mixed use neighborhood, common destinations, community centre, destinations, Destinations / services (overall/unspecific) access / availability , Education facilities access / availability, entertainment, exercise / gym facilities, food outlets, Food outlets access / availability, General services access / availability, Government / finance services access / availability, green space and recreational facilities, gym / fitness facilities, health and aged care, Health and aged care access / availability, institutional / industrial, land use mix - access, land use mix - destination diversity, other destinations, Other service / institution access / availability, outdoor sports field, park / open space, park/green space, parks, parks / open space / recreation, Parks / public open space, Parks / public open space access / availability, places of employment, playground, public transit, Public transport, Public transport access / availability, public transportation access, recreation areas, Recreation facilities access / availability, recreational facilities, religious, Religious institution access / availability, retail establishment, school, shopping mall, shops / commercial, shops / commercial / services, Shops / commercial access / availability, Social recreation facilities access / availability, swimming pool, Transit stops |
| Aesthetics & cleanliness / order | Aesthetically pleasing scenery, aesthetics, aesthetics and cleanliness/order, greenery, Greenery and aesthetically pleasing scenery, littering / vandalism / decay, littering / vandalism / decay / vacant buildings, pollution, pollution [air, noise, sewer] |
| Pedestrian / cycling infrastructure & streetscape | Barriers to walking / cycling , barriers to walking / cycling, benches / sitting facilities, Cycle / walk-friendly infrastructure, easy access to building entrance, easy access to building entrance, footpaths presence / quality, human or motorised traffic volume, indoor places for walking, Lives in weekend pedestrian-only corridor, No physical barriers to walking, other infrastructure for walking / cycling, paths, Pavement/footpath quality, pedestrian & cycling infrastructure; streetscape, pedestrian friendly features, public toilet, public toilets, sidewalk condition, sidewalk coverage, Sidewalk functionality, sidewalk presence, slopes / hilliness, Street lighting, street lights, trails, Walk-friendly infrastructure |
| Residential density / urbanisation | New urbanism, Residential density, residential density / urbanisation, residential households, sprawl, Urbanisation |
| Safety & traffic | Automobile traffic volume, crime / personal safety, general safety, human or motorised traffic volume, safety and traffic, Safety from traffic, traffic / pedestrian safety |
| Street connectivity | Connectivity, intersection frequency, street connectivity, street intersections |
| Walkability | Walkability |

| Table S4. Interpersonal determinant categorization | |
| --- | --- |
| **Categories** | **Determinants (terms) from reviews included in category** |
| General social support | General social support, network size, social engagement |
| Loneliness | Loneliness, loneliness-living alone |
| Social isolation | Social isolation |
| Social support for physical activity | Social support for physical activity – exercise group, social support for physical activity – family, social support for physical activity – friends, social support for physical activity – friends & family, social support for physical activity – friends, family, and doctors, social support for physical activity – friends, partner, and family |

| Table S5. Intrapersonal determinant categorization | |
| --- | --- |
| **Categories** | **Determinants (terms) from reviews included in category** |
| Age | Age |
| Balance | Balance, Berg Balance scale, |
| Body composition | Body mass index, body fat percentage, fat free mass |
| Cardiorespiratory fitness | Cardio respiratory fitness, Peak oxygen consumption, Six-Minute Walk Test, VO2 peak |
| Cognition | Cognition, global cognition, Montreal Cognitive Assessment, Short Portable Mental Status questionnaire, |
| Depression | Depression, Geriatric Depression scale, the Center for Epidemiological studies depression scale, Short Depression-Happiness scale, Beck Depression Inventory-ii, Hospital Anxiety and Depression scale, |
| Dizziness | Dizziness |
| Education | Education |
| Energy intake | Energy intake |
| Ethnicity | Ethnicity |
| Fatigue | Fatigue |
| Gender | Gender |
| History of falls | History of falls |
| Impairment | Impairment |
| Mobility | Gait speed and walk capacity, mobility, Short Physical Performance Battery, gait speed, activities of daily living function, Rivermead mobility index, Functional Independence measure |
| Number of comorbidities | Comorbidity (higher # of conditions) |
| Number of medications | Medications (>4) |
| Physical function | Physical function, lower extremity function, Rivermead motor assessment, global motor function, grip strength, lower limb strength, |
| Quality of life | Short form 36, EuroQOL-5D, health related quality of life, physical health related quality of life, |
| Resting metabolic rate | Resting metabolic rate |
| Self-efficacy - balance | Activities-specific balance confidence scale, balance self-efficacy |
| Self-efficacy - exercise | Short self-efficacy scale fore exercise |
| Self-efficacy - falls | Falls self-efficacy scale |
| Self-efficacy - general | General perceived self-efficacy scale |
| Sex | Sex |
| Sleep | Sleep |
| Social functioning | Social functioning |
| Socioeconomic status | Socioeconomic status |
| Subjective cognitive impairment | Subjective cognitive impairment |
| Waking hours in a day | Waking hours in a day |
| Determinants related to specific clinical populations | |
| Apathy | Apathy |
| Autonomic function | Autonomic symptom scale |
| Delirium | Delirium |
| Dementia – behavioural function | Neuropsychiatric inventory caregiver, neuropsychiatric inventory patient, revised memory and behavior problem checklist - disruption |
| Dementia – duration | Duration of dementia |
| Dementia - severity | Dementia severity |
| Left side infarct | Side of infarct |
| Quality of life - stroke | Stroke impact scale |
| Visual neglect | Presence of visual neglect |
| Years since stroke | Years since stroke |

# Physical activity definitions and inclusion summary

| Table S6. Physical activity definitions and inclusion | | | | |
| --- | --- | --- | --- | --- |
| Review author | PA type | Definition | Inclusion criteria | Definition reference |
| Barnett – 2017 [#] | Total PA, total MVPA, & walking | Not reported | Objectively measured or self-reported PA and/or walking that was not specific to a single PA domain only | None |
| Cerin – 2017 [#] | Active travel | Walking or cycling to a destination | A measure of engagement in, frequency and/or amount of walking and/or cycling for transport | None |
| Hong – 2008 [#] | Exercise program | Not reported | Excluded: “studies of multicomponent exercise, exercise stress test, single bout of exercise, exercise tolerance, drug study using exercise test | None |
| Lindsay Smith – 2017 [#] | PA | Not reported | PA was measured objectively or subjectively using measures with established validity as reported in the individual papers, or with clear face validity | None |
| Rosso – 2011[#] | Walking | Not reported | Outcomes included measures of mobility or disability and physical functioning as described in Verbrugge’s disablement model | None |
| Sun – 2013 [#] | PA | Not reported | Reported the proportion of any of PA recommendation or guidelines achieved by the sample | None |
| Van Cauwenberg – 2018 [#] | Leisure time PA | PA can be accumulated through engagement in various activity domains: work, household, transportation, and leisure | LTPA outcome (engagement in, frequency and/or amount of leisure-time walking, cycling and/or PA) | Sallis J, et al. 2006.^1^ |
| Yau – 2022 [#] | Exercise program | Not Reported | An exercise intervention that was delivered in the home or community setting that may or may not be supervised, for example, home-based programs, group-based programs, clinic-based programs, motivational interviewing, and telerehabilitation | None |
| Specific clinical populations in community dwelling settings | | | | |
| Stubbs – 2014 [#] | PA | Not reported | The dependent variable was a measure of PA participation | None |
| Thilarajah – 2018 [#] | PA | Any physical movement that causes energy expenditure because of skeletal muscle contraction | Explored associations with physical activity levels, studies where physical ability or function was measured instead, or where physical activity was not the dependent variable, were excluded | Caspersen CJ, 1985.^2^ |
| Wion – 2019 [#] | PA | Any bodily movement that requires energy (e.g., walking at a moderate pace or gardening) | Activity participation measure (specifically physical, social, or cognitive activities) | World Health Organization.^3^ |

# Physical activity outcome operationalization example

| Table S7. Examples of reported physical activity outcomes with respect to walking | | | |
| --- | --- | --- | --- |
|  | Dichotomous | Continuous | Categorical |
| Frequency | y/n (1+walks/day)  y/n (10+min/occurrence)  y/n (any/none) (frequency/week)  y/n (daily)  y/n (once/2days)  y/n (trips/2 days)  y/n (weekly frequency)  y/n (weekly participation)  1 walk trip/week | blocks/day days/week frequency  frequency (# of services accessed by walking)  frequency  frequency/duration  frequency/month  frequency/week times/week  trips/day  trips/week | 3 categories (walking trips frequency/day) |
| Time | y/n (<150 mins/week)  y/n (>0 mins/week)  y/n (150+ mins/week)  y/n (2.5+hours/week)  y/n (30+ mins/week)  y/n (30+mins/day*5 days/week)  y/n (5+days/week)  y/n (60+ min)  y/n (60+ mins/week)  y/n (likelihood>0 mins/week)  y/n (mins/week) | hours/day hours/week  mins/2 weeks mins/day  mins/last 2weeks  mins/week | 3 categories (0/10-149/150+) |
| Other | y/n (10,000 steps/day)  y/n (500+MET mins/week)  y/n (high walker)  y/n (likelihood)  y/n (non-walkers odds)  y/n (non-walkers)  y/n (some) | amount composite latent factor median kcal/week (25th, 75th %tile)  MET hours/week score  steps/day | 3 categories (inactive/low/active)  3 categories (low/moderate/high) |

# Relationship summary between determinants and adherence to exercise

| Table S8. Direction and pooled effects from meta-analytical results of relationships between determinants and exercise program adherence | | | | | |
| --- | --- | --- | --- | --- | --- |
|  | Yau 2022^a^ [#] (number of studies, total sample size) | Yau 2022^b^ [#] (number of studies, total sample size) | | Hong 2008^C^ [#] | |
| **Exercise program components** | | | | | |
| Aerobic | Null OR | - | | - | |
| Balance | Null OR | - | | - | |
| Flexibility | Null OR | - | | - | |
| Functional | Null OR | - | | - | |
| Strength | Null OR | - | | - | |
| **Exercise program characteristics** | | | | | |
| Duration  Less than 6 months  More than 6 months  Weeks | -OR  -OR  - | -  -OR  - | | -  -  -$\beta$ | |
| Format (group) | Null OR | - | | +$\beta$ | |
| Frequency  at least 3/weeks | -OR | Null OR | | - | |
| Setting  Home | Null OR | - | | - | |
| Supervised | +OR | +OR | | - | |
| Physiotherapist | Null OR | | - | | - |
| Abbreviations: *Null* OR odds ratio was not significant, *+OR* significant positive odds ratio, *-OR* significant negative odds ratio, + $\beta$ positive significant standardized coefficient, - $\beta$ negative significant standardized coefficient, *IC* inconsistent results,  (a) Univariable meta-analysis  (b) Multivariate meta-analysis  (c) Multivariate Meta-regression | | | | | |

#

# Summary of JBI Critical Appraisal Checklist

**
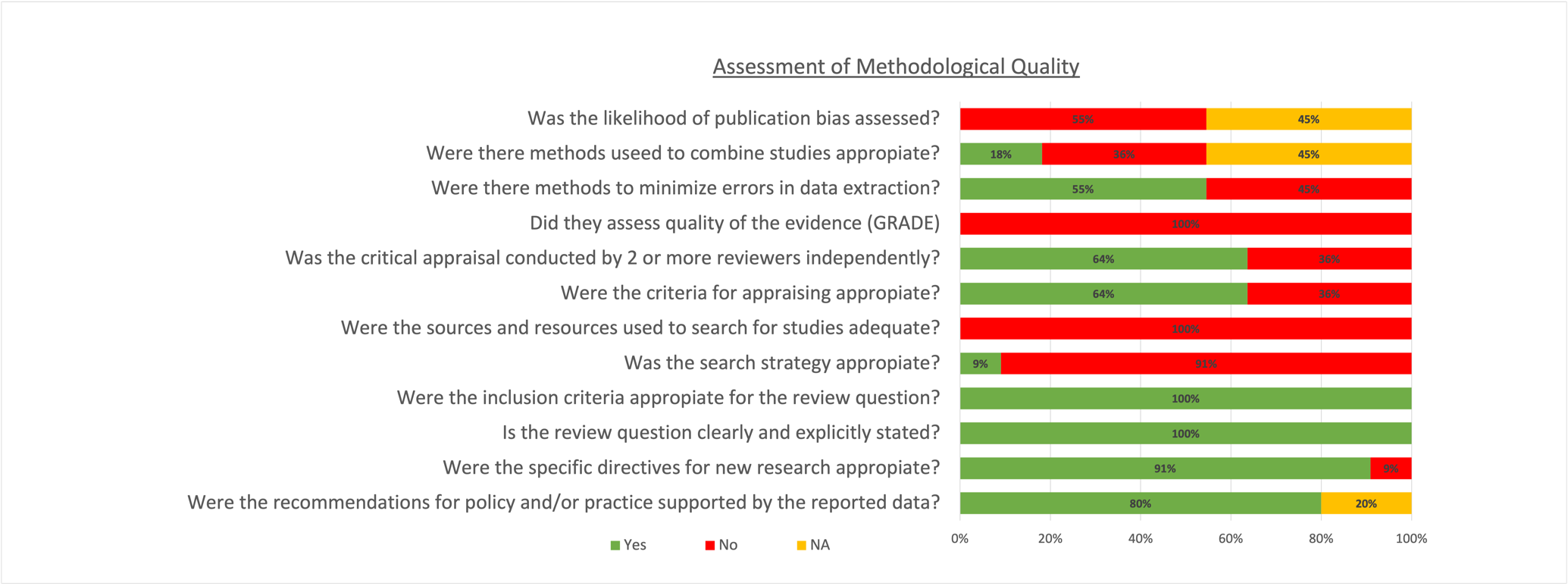
**

Figure S1. Summary of JBI Critical Appraisal Checklist for the eleven included reviews

# Additional self-report instrument summary

| Box S1. Reported Items or scales from Cohort studies (n=19) |
| --- |
| Behavioral Risk Factor Surveillance System (BRFSS)  California Health Interview survey questionnaire (CHIS)  Canadian Community Health survey questionnaire (CCHS)  EpiPorto physical activity questionnaire  General Social Survey Time Use questionnaire  Harvard Alumni Health questionnaire  Health Interview survey 1985  Health interview survey items  Longitudinal Aging Study Amsterdam (LASA) Physical activity questionnaire  National Health Interview Survey (NHIS)  Netherlands Housing survey questionnaire  New South Wales Falls Prevention survey questionnaire (NSW)  New South Wales Older People's Health Survey (NSW)  Nurses' Health Study questionnaire  Oslo Health Study questionnaire  Senior Health and Physical Exercise questionnaire  Study on global AGEing and adult health (SAGE)  Women's Health Initiative questionnaire (WHI)  Zhongshan Household Travel survey questionnaire |

# Socioecological model

Model was based on stokols et all (1996), Mcleroy et al (1988), and jones et al (2018).^4-6^

| **LEVELS** | Description |
| --- | --- |
| **Intrapersonal (Individual)** | Sociodemographic, biological/physiological/function, psychosocial |
| **Interpersonal (social environment)** | Formal and informal personal relationships, contacts (inner social circle) |
| **Organizational** | Rules and regulations around a behaviour, benefits or incentives |
| **Community** | Context for behaviour: social cultural norms (Outer social circle, beliefs of these groups results in different norms), physical environment (natural & built) |
| **Public Policy** | Policies that restrict behaviour, provide incentives, allocation of grants, restriction of allocation of resources |

1. Sallis JF, Cervero RB, Ascher W, Henderson KA, Kraft MK, Kerr J. An ecological approach to creating active living communities. Annu Rev Public Health. 2006;27:297-322.

2. Caspersen CJ, Powell KE, Christenson GM. Physical Activity Exercise and Physical Fitness Definitions and Distinctions for Health Related Research. Public Health Rep. 1985;100(2):126-31.

3. World Health Organization. Physical Activity [Fact sheet] (2018. February 23) [Available from: <https://www.who.int/news-room/fact-sheets/detail/physical-activity>.

4. Jones GR, Stathokostas L, Young BW, Wister AV, Chau S, Clark P, et al. Development of a physical literacy model for older adults – a consensus process by the collaborative working group on physical literacy for older Canadians. BMC Geriatr. 2018;18(1):13.

5. Stokols D. Translating Social Ecological Theory into Guidelines for Community Health Promotion. Am J Health Promot. 1996;10(4):282-98.

6. McLeroy KR, Bibeau D, Steckler A, Glanz K. An ecological perspective on health promotion programs. Health Educ Q. 1988;15(4):351-77.

# Full-text Exclusion Reasons

| Table S9. Full-text Exclusion Reason | | | | |
| --- | --- | --- | --- | --- |
| Year | Authors | Title | Exclusion Reason | Comments |
| 1992 | Mullen, P. D.; Mains, D. A.; Velez, R. | A meta-analysis of controlled trials of cardiac patient education | Determinant | not looking at |
| 2001 | Kruger, Judy | Meta-analysis of factors related to self-efficacy for exercise in older adults | Determinant | reverse relationship |
| 2001 | Martin, Kathleen A.; and Sinden, Adrienne R. | Who will stay and who will go? A review of older adults' adherence to randomized controlled trials of exercise | Age | Wrong age group |
| 2002 | Jones, M. M.; and Haight, B. K. | Environmental transformations: an integrative review | Age | could not confirm age |
| 2002 | Lewis, B. A. et al. | Psychosocial mediators of physical activity behavior among adults and children | Age | could not confirm age |
| 2002 | Trost, S. G. et al. | Correlates of adults' participation in physical activity: review and update | Age | Wrong age group |
| 2003 | Plonczynski, D. J. | Physical activity determinants of older women: what influences activity? | Physical activity | No PA outcome |
| 2004 | Cunningham, G. O.; and Michael, Y. L. | Concepts guiding the study of the impact of the built environment on physical activity for older adults: a review of the literature | Age | could not confirm age |
| 2004 | Fischbacher, C. M.; Hunt, S.; Alexander, L. | How physically active are South Asians in the United Kingdom? A literature review | Age | Wrong age group |
| 2005 | Gidlow, C. et al. | Attendance of exercise referral schemes in the UK: a systematic review | Age | Wrong age group |
| 2005 | Hong, Seung-youn | Predictors of exercise adherence in sedentary older adults: systematic meta-analytic approach | Wrong study design |  |
| 2005 | Matson-Koffman, D. M. et al. | A site-specific literature review of policy and environmental interventions that promote physical activity and nutrition for cardiovascular health: what works? | Age | Wrong age group |
| 2006 | Allender, S.; Cowburn, G.; Foster, C. | Understanding participation in sport and physical activity among children and adults: a review of qualitative studies | Physical activity | No physical activity outcome |
| 2006 | Gidlow, C. et al. | A systematic review of the relationship between socio-economic position and physical activity | Age | Wrong age group |
| 2007 | Mendonza, M.; Patel, H.; Bassett, S. | Influences of psychological factors and rehabilitation adherence on the outcome post anterior cruciate ligament injury/surgical reconstruction | Age | Wrong age group |
| 2007 | Tucker, P.; and Gilliland, J. | The effect of season and weather on physical activity: a systematic review | Age | could not extract older adults |
| 2007 | Tzormpatzakis, N.; and Sleap, M. | Participation in physical activity and exercise in Greece: a systematic literature review | Age | could not extract older adults |
| 2007 | Wendel-Vos, W. et al. | Potential environmental determinants of physical activity in adults: a systematic review | Age | could not extract older adults |
| 2008 | Dahan-Oliel, N.; Gelinas, I.; Mazer, B. | Social participation in the elderly: What does the literature tell us? | Physical activity | No PA outcome |
| 2008 | Drygas, W. et al. | Increasing recreational and leisure time physical activity in Poland - How to overcome barriers of inactivity | Wrong study design |  |
| 2009 | Agrimson, L. B.; and Taft, L. B. | Spiritual crisis: a concept analysis | Wrong study design |  |
| 2009 | Booth, J. et al. | The effects of lifestyle and behavioural interventions for urinary incontinence on mobility, physical activity and falls in older people: A comprehensive systematic review | Wrong study design |  |
| 2009 | Daniel, K. et al. | What are the social consequences of stroke for working-aged adults? A systematic review | Age | Wrong age group |
| 2009 | Ekkekakis, P. | Let them roam free? Physiological and psychological evidence for the potential of self-selected exercise intensity in public health | Age | could not extract older adults |
| 2009 | Hagger, M. S.; and Chatzisarantis, N. L. | Integrating the theory of planned behaviour and self-determination theory in health behaviour: a meta-analysis | Age | could not extract older adults |
| 2009 | Keller, C. et al. | Moderators of physical activity in Hispanic women | Age | could not extract older adults |
| 2009 | Tudor-Locke, C.; Hart, T. L.; Washington, T. L. | Expected values for pedometer-determined physical activity in older populations | Determinant | not looking at |
| 2009 | Tudor-Locke, Catrine; Hart, Teresa L.; Washington, Tracy L. | Correction: Expected values for pedometer-determined physical activity in older populations | Determinant | not looking at |
| 2010 | Arnould, Benoit et al. | The impact of herpes zoster and subsequent chronic pain on patients' daily lives | Age | could not confirm age |
| 2010 | Beard, J. R.; and Petitot, C. | Ageing and urbanization: Can cities be designed to foster active ageing? | Age | Wrong age group |
| 2010 | Jack, K. et al. | Barriers to treatment adherence in physiotherapy outpatient clinics: a systematic review | Physical activity | No PA outcome |
| 2010 | Weih, M. et al. | Physical activity and alzheimer's disease: A meta-analysis of cohort studies | Determinant | reverse relationship |
| 2011 | Baert, V. et al. | Motivators and barriers for physical activity in the oldest old: a systematic review | Physical activity | No PA outcome |
| 2011 | Bowen, Robert S.; Turner, Michael J.; Lightfoot, J. Timothy | Sex Hormone Effects on Physical Activity Levels: Why Doesn't Jane Run as Much as Dick? | Wrong study design |  |
| 2011 | Bragaru, Mihail et al. | Amputees and Sports | Physical activity | No PA outcome |
| 2011 | Koeneman, M. A. et al. | Determinants of physical activity and exercise in healthy older adults: A systematic review | Duplicate |  |
| 2011 | Koeneman, M. A. et al. | Determinants of physical activity and exercise in healthy older adults: a systematic review | Age | Wrong age group |
| 2011 | Leavy, J. E. et al. | Physical activity mass media campaigns and their evaluation: a systematic review of the literature 2003-2010 | Age | could not confirm age |
| 2011 | McLeod, K. M.; and Johnson, C. S. | A systematic review of osteoporosis health beliefs in adult men and women | Physical activity | No PA outcome |
| 2011 | Siddiqi, Z.; Tiro, J. A.; Shuval, K. | Understanding impediments and enablers to physical activity among African American adults: a systematic review of qualitative studies | Age | could not extract older adults |
| 2011 | Taylor, Gavin H.; Wilson, Sarah L.; Sharp, John | Medical, psychological, and sociodemographic factors associated with adherence to cardiac rehabilitation programs: a systematic review | Physical activity | No PA outcome |
| 2011 | Tiemey, Stephanie et al. | What Can We Learn From Patients With Heart Failure About Exercise Adherence? A Systematic Review of Qualitative Papers | Physical activity | No physical activity outcome |
| 2011 | Tierney, S. et al. | Understanding barriers and enablers of physical activity among patients with heart failure: A systematic review of qualitative studies | Duplicate |  |
| 2011 | Toohey, A. M.; and Rock, M. J. | Unleashing their potential: a critical realist scoping review of the influence of dogs on physical activity for dog-owners and non-owners | Age | could not extract older adults |
| 2011 | Van Cauwenberg, J. et al. | Relationship between the physical environment and physical activity in older adults: a systematic review | Setting | Not specified |
| 2011 | Vorrink, S. N. et al. | Level of daily physical activity in individuals with COPD compared with healthy controls | Age | Wrong age group |
| 2012 | Barber, F. D. | Social support and physical activity engagement by cancer survivors | Age | Wrong age group |
| 2012 | Barnett, I.; Guell, C.; Ogilvie, D. | The experience of physical activity and the transition to retirement: a systematic review and integrative synthesis of qualitative and quantitative evidence | Physical activity | No PA outcome |
| 2012 | Barnett, I.; van Sluijs, E. M.; Ogilvie, D. | Physical activity and transitioning to retirement: a systematic review | Physical activity | No physical activity outcome |
| 2012 | Beenackers, Marielle A. et al. | Socioeconomic inequalities in occupational, leisure-time, and transport related physical activity among European adults: A systematic review | Age | could not extract older adults |
| 2012 | Engberg, E. et al. | Life events and change in leisure time physical activity: a systematic review | Age | could not extract older adults |
| 2012 | Engberg, Elina et al. | Life Events and Change in Leisure Time Physical Activity | Duplicate |  |
| 2012 | Gerber, Markus; Barker, Dean; Puhse, Uwe | Acculturation and physical activity among immigrants: A systematic review | Age | Wrong age group |
| 2012 | Griffin, D. W.; Harmon, D. C.; Kennedy, N. M. | Do patients with chronic low back pain have an altered level and/or pattern of physical activity compared to healthy individuals? A systematic review of the literature | Setting | Not specified |
| 2012 | Hand, C. et al. | Neighborhood influences on participation among older adults with chronic health conditions: A scoping review | Physical activity | No PA outcome |
| 2012 | Horne, M.; and Tierney, S. | What are the barriers and facilitators to exercise and physical activity uptake and adherence among South Asian older adults: a systematic review of qualitative studies | Physical activity | No PA outcome |
| 2012 | Munsterman, T.; Takken, T.; Wittink, H. | Are persons with rheumatoid arthritis deconditioned? A review of physical activity and aerobic capacity | Age | Wrong age group |
| 2012 | Palmer, K. T. | The older worker with osteoarthritis of the knee | Wrong study design |  |
| 2012 | Pavey, T. et al. | Levels and predictors of exercise referral scheme uptake and adherence: a systematic review | Age | could not extract older adults |
| 2012 | Puts, M. T. et al. | A systematic review of unmet needs of newly diagnosed older cancer patients undergoing active cancer treatment | Physical activity | No PA outcome |
| 2012 | Leirós-Rodríguez, J. L.; and García, Soidán | Geriatric revitalization programs: possibilities of the public parks to promote healthy physical activity in the elderly A review of the literature | Age | could not confirm age |
| 2012 | Teixeira, Pedro J. et al. | Exercise, physical activity, and self-determination theory: A systematic review | Age | could not extract older adults |
| 2013 | Ardern, C. L. et al. | A systematic review of the psychological factors associated with returning to sport following injury | Age | Wrong age group |
| 2013 | Barbic, S. P.; Bartlett, S. J.; Mayo, N. E. | Emotional vitality: Concept of importance for rehabilitation | Physical activity | No PA outcome |
| 2013 | Boehm, J. et al. | Barriers and motivators to exercise for older adults: a focus on those living in rural and remote areas of Australia | Wrong study design |  |
| 2013 | Christian, H. E. et al. | Dog ownership and physical activity: a review of the evidence | Age | could not extract older adults |
| 2013 | Grasser, G. et al. | Objectively measured walkability and active transport and weight-related outcomes in adults: a systematic review | Age | Wrong age group |
| 2013 | McGrath, Colleen E.; and Rudman, Debbie Laliberte | Factors that influence the occupational engagement of older adults with low vision: a scoping review | Age | could not confirm age |
| 2013 | Morin Fraile, V. | [Promotion of physical activity] | Unable to locate full text |  |
| 2013 | Neto, P. et al. | Ability to work in the elderly: Integrative literature review | Physical activity | No PA outcome |
| 2013 | Olsen, Jeanette M. | An integrative review of literature on the determinants of physical activity among rural women | Age | Wrong age group |
| 2013 | Perry, M. | Factors contributing to youth and adult dropout from organised sport and physical activity | Unable to locate full text |  |
| 2013 | Purnell, T. S. et al. | Comparison of life participation activities among adults treated by hemodialysis, peritoneal dialysis, and kidney transplantation: a systematic review | Physical activity | No PA outcome |
| 2013 | Rhodes, R. E.; and Dickau, L. | Moderators of the intention-behaviour relationship in the physical activity domain: a systematic review | Age | Wrong age group |
| 2013 | Rosenkranz, Richard R. et al. | A review of enablers and barriers to physical activity participation among older people of New Zealand and international populations | Physical activity | No physical activity outcome |
| 2013 | Stubbs, B et al. | Are older adults with chronic musculoskeletal pain less active than older adults without pain? A systematic review and meta-analysis | Setting | Less than 80% community-dwelling |
| 2014 | Al Abed, Naser A.; Davidson, Patricia M.; Hickman, Louise D. | Healthcare needs of older Arab migrants: a systematic review | Physical activity | No physical activity outcome |
| 2014 | Anderiesen, H et al. | A systematic review--physical activity in dementia: the influence of the nursing home environment | Setting | Not community-dwelling |
| 2014 | Cunha Loureiro, Ana Paula et al. | A Review of the Relationship Between Poststroke Fatigue and Physical Activity | Age | could not confirm age |
| 2014 | English, C. et al. | Physical activity and sedentary behaviors in people with stroke living in the community: a systematic review | Age | Wrong age group |
| 2014 | Fereshtehnejad, Seyed-Mohammad and Lökk, Johan | Active Aging for Individuals with Parkinson's Disease: Definitions, Literature Review, and Models | Wrong study design |  |
| 2014 | Gimeno-Santos, E. et al. | Determinants and outcomes of physical activity in patients with COPD: a systematic review | Age | Wrong age group |
| 2014 | Han, B. et al. | How much neighborhood parks contribute to local residents' physical activity in the City of Los Angeles: a meta-analysis | Wrong study design |  |
| 2014 | Heiss, Valerie; and Petosa, Rick | Correlates of Physical Activity Among Adults With Type 2 Diabetes: A Systematic Literature Review | Age | Wrong age group |
| 2014 | Jaarsma, E. A.; Dijkstra, P. U.; Geertzen, J. H. B.; Dekker, R. | Barriers to and facilitators of sports participation for people with physical disabilities: A systematic review | Age | Wrong age group |
| 2014 | Kampshoff, C. S. et al. | Determinants of exercise adherence and maintenance among cancer survivors: a systematic review | Age | could not extract older adults |
| 2014 | Larkin, L. and Kennedy, N. | Correlates of physical activity in adults with rheumatoid arthritis: a systematic review | Age | could not extract older adults |
| 2014 | Moran, M. et al. | Understanding the relationships between the physical environment and physical activity in older adults: a systematic review of qualitative studies | Physical activity | No PA outcome |
| 2014 | O'Driscoll, T. et al. | A systematic literature review of sport and physical activity participation in culturally and linguistically diverse (CALD) migrant populations | Age | Wrong age group |
| 2014 | Perrier-Melo, Raphael José  et al. | Active Video Games, balance and energy expenditure in elderly:a systematic review | Determinant | not looking at |
| 2014 | Picorelli, A et al. | A systematic review--physical activity in dementia: the influence of the nursing home environment | Setting | Not specified |
| 2014 | Stahl, S. T.; and Schulz, R. | Changes in routine health behaviors following late-life bereavement: a systematic review | Age | could not extract older adults |
| 2014 | Stults-Kolehmainen, M. A.; and Sinha, R. | The effects of stress on physical activity and exercise | Age | could not extract older adults |
| 2014 | Sze Loon, Chow; Anselm Su, Ting; Tin Tin, S. U. | Development of Conceptual Framework to Understand Factors Associated with Return to Work among Cancer Survivors: A Systematic Review | Age | could not extract older adults |
| 2014 | Westgarth, C.; Christley, R. M.; Christian, H. E. | How might we increase physical activity through dog walking?: A comprehensive review of dog walking correlates | Age | Wrong age group |
| 2014 | Yen, Irene H. et al. | How Design of Places Promotes or Inhibits Mobility of Older Adults: Realist Synthesis of 20 Years of Research | Age | could not extract older adults |
| 2015 | Akande, V. O. et al. | Determinants of dietary behavior and physical activity among Canadian Inuit: a systematic review | Age | Wrong age group |
| 2015 | Bancroft, C. et al. | Association of proximity and density of parks and objectively measured physical activity in the United States: A systematic review | Age | could not extract older adults |
| 2015 | Boqin, Xie; and Arslanian-Engoren, Cynthia | Self-Concepts of Exercise in Frail Older Adults With Heart Failure: A Literature Review | Physical activity | No PA outcome |
| 2015 | Denkinger, M. D. et al. | Factors associated with fear of falling and associated activity restriction in community-dwelling older adults: a systematic review | Physical activity | No PA outcome |
| 2015 | Elhakeem, Ahmed et al. | Childhood socioeconomic position and adult leisure-time physical activity: a systematic review | Age | Wrong age group |
| 2015 | Farrance, C.; Tsofliou, F.; Clark, C. J. | Adherence to community based group exercise interventions in older people: A systematic review | Duplicate |  |
| 2015 | Farrance, C.; Tsofliou, F.; Clark, C. J. | Evaluating the views of participants and adherence rates of community based group exercise interventions: A mixed methods systematic review | Determinant | not looking at |
| 2015 | Farrance, C.; Tsofliou, F.; Clark, C. J. | Understanding the views of older people in adhering to community based group exercise interventions: A qualitative systematic review | Duplicate |  |
| 2015 | Farren, L. et al. | Mall Walking Program Environments, Features, and Participants: A Scoping Review | Physical activity | No PA outcome |
| 2015 | Franco, M. R. et al. | Older people's perspectives on participation in physical activity: a systematic review and thematic synthesis of qualitative literature | Physical activity | No physical activity outcome |
| 2015 | Hajna, S. et al. | Associations between neighbourhood walkability and daily steps in adults: a systematic review and meta-analysis | Age | could not extract older adults |
| 2015 | Hamilton, M. et al. | Predictors of physical activity levels of individuals following traumatic brain injury (TBI) remain unclear: A systematic review | Age | Wrong age group |
| 2015 | Park, C. L.; Braun, T.; Siegel, T. | Who practices yoga? A systematic review of demographic, health-related, and psychosocial factors associated with yoga practice | Age | could not confirm age |
| 2015 | Stubbs, B.; Hurley, M.; Smith, T. | What are the factors that influence physical activity participation in adults with knee and hip osteoarthritis? A systematic review of physical activity correlates | Age | could not extract older adults |
| 2016 | Allen, L. et al. | Poverty and risk factors for non-communicable diseases in developing countries: A systematic review | Duplicate |  |
| 2016 | Anita, E. W. et al. | A systematic review of consumer perceived health service needs related to osteoarthritis | Physical activity | No PA outcome |
| 2016 | Arnold, John B.; Walters, Julie L.; Ferrar, Katia E. | Does Physical Activity Increase After Total Hip or Knee Arthroplasty for Osteoarthritis? A Systematic Review | Age | Wrong age group |
| 2016 | Backonja, U. et al. | Visualization approaches to support healthy aging: A systematic review | Physical activity | No PA outcome |
| 2016 | Baxter, S. et al. |  | Wrong study design |  |
| 2016 | Benton, J. S. et al. | The effect of changing the built environment on physical activity: a quantitative review of the risk of bias in natural experiments | Wrong study design |  |
| 2016 | Dairo, Y. M. et al. | Physical activity levels in adults with intellectual disabilities: A systematic review | Age | Wrong age group |
| 2016 | Day, K. | Built environmental correlates of physical activity in China: A review | Age | could not extract older adults |
| 2016 | Devereux-Fitzgerald, A. et al. | The acceptability of physical activity interventions to older adults: A systematic review and meta-synthesis | Physical activity | No PA outcome |
| 2016 | Dobson, F. et al. | Barriers and Facilitators to Exercise Participation in People with Hip and/or Knee Osteoarthritis: Synthesis of the Literature Using Behavior Change Theory | Age | Wrong age group |
| 2016 | Dobson, Fiona et al. | Barriers and Facilitators to Exercise Participation in People with Hip and/or Knee Osteoarthritis | Physical activity | No PA outcome |
| 2016 | Evenson, K. R. et al. | Park characteristics, use, and physical activity: A review of studies using SOPARC (System for Observing Play and Recreation in Communities) | Wrong study design |  |
| 2016 | Klouche, Shahnaz et al. | Return to Sport After Rotator Cuff Tear Repair | Determinant | not looking at |
| 2016 | Klugar, M. et al. | The personal active aging strategies of older adults in Europe: a systematic review of qualitative evidence | Physical activity | No PA outcome |
| 2016 | Learmonth, Y. C.; and Motl, R. W. | Physical activity and exercise training in multiple sclerosis: a review and content analysis of qualitative research identifying perceived determinants and consequences | Age | could not extract older adults |
| 2016 | Mabry, R. et al. | A systematic review of physical activity and sedentary behaviour research in the oil-producing countries of the Arabian Peninsula | Age | Wrong age group |
| 2016 | McDermott, Máirtín S. et al. | The moderating impact of temporal separation on the association between intention and physical activity: a meta-analysis | Age | could not extract older adults |
| 2016 | Morgan, F. et al. | Adherence to exercise referral schemes by participants - what do providers and commissioners need to know? A systematic review of barriers and facilitators | Physical activity | No PA outcome |
| 2016 | Papageorgiou, Nicole et al. | Occupational therapy and occupational participation in community dwelling older adults: A review of the evidence | Determinant | reverse relationship |
| 2016 | Pels, Fabian; and Kleinert, Jens | Loneliness and physical activity: A systematic review | Age | Wrong age group |
| 2016 | Ruano-Ravina, A. et al. | Participation and adherence to cardiac rehabilitation programs. A systematic review | Age | could not confirm age |
| 2016 | Saunders, T. et al. | Objectively Measured Steps/Day in Patients With Chronic Obstructive Pulmonary Disease: A Systematic Review and Meta-Analysis | Age | Wrong age group |
| 2016 | Streber, R.; Peters, S.; Pfeifer, K. | Systematic Review of Correlates and Determinants of Physical Activity in Persons With Multiple Sclerosis | Age | Wrong age group |
| 2016 | Stubbs, Brendon et al. | How much physical activity do people with schizophrenia engage in? A systematic review, comparative meta-analysis and meta-regression | Age | Wrong age group |
| 2016 | van Alphen, H. J.; Hortobagyi, T.; van Heuvelen, M. J. | Barriers, motivators, and facilitators of physical activity in dementia patients: A systematic review | Physical activity | No PA outcome |
| 2016 | van der Vorst, A. et al. | Limitations in Activities of Daily Living in Community-Dwelling People Aged 75 and Over: A Systematic Literature Review of Risk and Protective Factors | Physical activity | No PA outcome |
| 2016 | Vancampfort, D. et al. | Physical activity and sedentary behavior in people with bipolar disorder: A systematic review and meta-analysis | Age | Wrong age group |
| 2016 | Vancampfort, D. et al. | Physical Activity in People With Posttraumatic Stress Disorder: A Systematic Review of Correlates | Age | could not extract older adults |
| 2016 | Vaughan, M. et al. | Which Features of the Environment Impact Community Participation of Older Adults? A Systematic Review and Meta-Analysis | Physical activity | No PA outcome |
| 2016 | Winterbotham, Sonya; and du Preez, Jan | Psychosocial wellbeing in active older adults: A systematic review of qualitative literature | Determinant | not looking at |
| 2016 | Witjes, Suzanne et al. | Return to Sports and Physical Activity After Total and Unicondylar Knee Arthroplasty: A Systematic Review and Meta-Analysis | Physical activity | No PA outcome |
| 2016 | Won, J.; Lee, C.; Forjuoh, S. N.; Ory, M. G. | Neighborhood safety factors associated with older adults' health-related outcomes: A systematic literature review | Age | Wrong age group |
| 2016 | Zapata-Diomedi, B.; and Veerman, J. L. | The association between built environment features and physical activity in the Australian context: a synthesis of the literature | Age | could not extract older adults |
| 2017 | Agmon, M.; Lavie, L.; Doumas, M. | The Association between Hearing Loss, Postural Control, and Mobility in Older Adults: A Systematic Review | Physical activity | No physical activity outcome |
| 2017 | Allen, L. et al. | Socioeconomic status and non-communicable disease behavioural risk factors in low-income and lower-middle-income countries: a systematic review | Age | Wrong age group |
| 2017 | Baldwin, Claire et al. | Accelerometry Shows Inpatients With Acute Medical or Surgical Conditions Spend Little Time Upright and Are Highly Sedentary: Systematic Review | Determinant | not looking at |
| 2017 | Bell, S et al. | Sexual Activity After 60: A Systematic Review of Associated Factors | Physical activity | No PA outcome |
| 2017 | Bertrand, Kim et al. | Walking Aids for Enabling Activity and Participation: A Systematic Review | Physical activity | No PA outcome |
| 2017 | Burgess, E.; Hassmen, P.; Pumpa, K. L. | Determinants of adherence to lifestyle intervention in adults with obesity: a systematic review | Age | Wrong age group |
| 2017 | Burton, R et al. | Motivators and Barriers for Older People Participating in Resistance Training: A Systematic Review | Physical activity | No PA outcome |
| 2017 | Cabilan, C. J.; and Hines, S. | The short-term impact of colorectal cancer treatment on physical activity, functional status and quality of life: a systematic review | Age | could not extract older adults |
| 2017 | Casey, B. et al. | Modifiable Psychosocial Constructs Associated With Physical Activity Participation in People With Multiple Sclerosis: A Systematic Review and Meta-Analysis | Age | Wrong age group |
| 2017 | Catt, Susan et al. | Patient-reported outcome measures of the impact of cancer on patients' everyday lives: a systematic review | Physical activity | No PA outcome |
| 2017 | De Pinho Bailon Almeida, Leidiane et al. | El anciano en centros de convivencia y ocio | Physical activity | No PA outcome |
| 2017 | Dolezal, B. A. et al. | Interrelationship between Sleep and Exercise: A Systematic Review | Determinant | reverse relationship |
| 2017 | Eisenberg, Y.; Vanderbom, K. A.; Vasudevan, V. | Does the built environment moderate the relationship between having a disability and lower levels of physical activity? A systematic review | Age | could not extract older adults |
| 2017 | Elhakeem, A. et al. | Intergenerational social mobility and leisure-time physical activity in adulthood: a systematic review | Age | Wrong age group |
| 2017 | Essery, R. et al. | Predictors of adherence to home-based physical therapies: a systematic review | Age | could not extract older adults |
| 2017 | Hannan, M.; and Bronas, U. G. | Barriers to exercise for patients with renal disease: an integrative review | Age | Wrong age group |
| 2017 | Jenkin, C. R. et al. | Sport and ageing: a systematic review of the determinants and trends of participation in sport for older adults | Physical activity | No PA outcome |
| 2017 | Kabisch, N.; van den Bosch, M.; Lafortezza, R. | The health benefits of nature-based solutions to urbanization challenges for children and the elderly - A systematic review | Age | could not confirm age |
| 2017 | Notthoff, N.; Reisch, P.; Gerstorf, D. | Individual Characteristics and Physical Activity in Older Adults: A Systematic Review | Setting | Not specified |
| 2017 | Pollard, T. M.; and Wagnild, J. M. | Gender differences in walking (for leisure, transport and in total) across adult life: a systematic review | Setting | Not specified |
| 2017 | Roberts, Christine E. et al. | Effect of Different Types of Physical Activity on Activities of Daily Living in Older Adults: Systematic Review and Meta-Analysis | Physical activity | No PA outcome |
| 2017 | Rodrigues, I. B. et al. | Facilitators and barriers to exercise adherence in patients with osteopenia and osteoporosis: a systematic review | Age | could not extract older adults |
| 2017 | Sandlund, M. et al. | Gender perspectives on views and preferences of older people on exercise to prevent falls: a systematic mixed studies review | Physical activity | No PA outcome |
| 2017 | Scarapicchia, Tanya Maria Filomena et al. | Social support and physical activity participation among healthy adults: a systematic review of prospective studies | Age | Wrong age group |
| 2017 | Sweeney, A. M.; and Culcea, I. | Does a future-oriented temporal perspective relate to body mass index, eating, and exercise? A meta-analysis | Age | could not extract older adults |
| 2017 | Tinker, Anthea et al. | Is exercise helpful for women aged 50 and over with mental health problems and what are the barriers to exercise? | Age | Wrong age group |
| 2017 | Tint, A.; Thomson, K.; Weiss, J. A. | A systematic literature review of the physical and psychosocial correlates of Special Olympics participation among individuals with intellectual disability | Age | Wrong age group |
| 2017 | van Buul, A. R. et al. | Association between morning symptoms and physical activity in COPD: a systematic review | Age | Wrong age group |
| 2017 | Withers, Thomas M. et al. | Is there a difference in physical activity levels in patients before and up to one year after unilateral total hip replacement? A systematic review and meta-analysis | Age | Wrong age group |
| 2018 | Al-Sari, U. A.; Tobias, J. H.; Clark, E. M. | Self-reported everyday physical activities in older people with osteoporotic vertebral fractures: a systematic review and meta-analysis | Physical activity | No physical activity outcome |
| 2018 | Al-Sari, U. A.; Tobias, J.; Clark, E. M. | Everyday physical activities in older people with compression vertebral fractures (VFs): A systematic review and meta-analysis | Unable to locate full text |  |
| 2018 | Amireault, S.; Baier, J. M.; Spencer, J. R. | Physical Activity Preferences Among Older Adults: A Systematic Review | Physical activity | No physical activity outcome |
| 2018 | An, R.; Zhang, S.; Ji, M.; Guan, C. | Impact of ambient air pollution on physical activity among adults: a systematic review and meta-analysis | Age | Wrong age group |
| 2018 | Arnold, Susan et al. | International perspective on factors influencing the performance of housework: a scoping review | Age | Wrong age group |
| 2018 | Bentley, A. J.; and Kelechi, T. J. | Motivators and Barriers to Walking in Older Adults With Peripheral Artery Disease | Physical activity | No physical activity outcome |
| 2018 | Bigonnesse, Catherine et al. | The role of neighborhood physical environment on mobility and social participation among people using mobility assistive technology | Physical activity | No physical activity outcome |
| 2018 | Cheval, B. et al. | Behavioral and Neural Evidence of the Rewarding Value of Exercise Behaviors: A Systematic Review | Age | Wrong age group |
| 2018 | Congello, N. C.; and Koniak-Griffin, D. | Review: Partner Support and Physical Activity among Mexican American Women | Age | Wrong age group |
| 2018 | Cordova-Rivera, L. et al. | A Systematic Review of Associations of Physical Activity and Sedentary Time with Asthma Outcomes | Age | Wrong age group |
| 2018 | Curtis, Amy et al. | Systematic review of the impact of arts for health activities on health, wellbeing and quality of life of older people living in care homes | Physical activity | No PA outcome |
| 2018 | de Lacy-Vawdon, C. J. et al. | Facilitators of Attendance and Adherence to Group-Based Physical Activity for Older Adults: A Literature Synthesis | Age | Wrong age group |
| 2018 | Edwards, N.; and Dulai, J. | Examining the relationships between walkability and physical activity among older persons: what about stairs? | Age | Wrong age group |
| 2018 | Gadais, T. et al. | Environments favorable to healthy lifestyles: A systematic review of initiatives in Canada | Age | Wrong age group |
| 2018 | Graham, Hilary et al. | The experiences of everyday travel for older people in rural areas: A systematic review of UK qualitative studies | Physical activity | No PA outcome |
| 2018 | Hoorntje, Alexander et al. | The Effect of Total Hip Arthroplasty on Sports and Work Participation: A Systematic Review and Meta-Analysis | Determinant | not looking at |
| 2018 | Lee, J. L. C.; Lo, T. L. T.; Ho, R. T. H. | Understanding Outdoor Gyms in Public Open Spaces: A Systematic Review and Integrative Synthesis of Qualitative and Quantitative Evidence | Age | could not confirm age |
| 2018 | Levy-Storms, L.; Chen, L.; Loukaitou-Sideris, A. | Older Adults' Needs and Preferences for Open Space and Physical Activity in and Near Parks: A Systematic Review | Physical activity | No PA outcome |
| 2018 | Liangruenrom, N. et al. | Physical activity and sedentary behaviour research in Thailand: a systematic scoping review | Age | could not extract older adults |
| 2018 | Lim, Y. M.; Kim, H.; Cha, Y. J. | Effects of environmental modification on activities of daily living, social participation and quality of life in the older adults: a meta-analysis of randomized controlled trials | Physical activity | No PA outcome |
| 2018 | Mathew Joseph, N.; Ramaswamy, P.; Wang, J. | Cultural factors associated with physical activity among U.S. adults: An integrative review | Age | Wrong age group |
| 2018 | McGowan, Laura J. et al. | How acceptable do older adults find the concept of being physically active? A systematic review and meta-synthesis | Physical activity | No PA outcome |
| 2018 | Mol, A et al. | Orthostatic hypotension and physical functioning in older adults: A systematic review and meta-analysis | Setting | Less than 80% community-dwelling |
| 2018 | Nam, Y. S.; Lee, G.; Yun, J. M.; Cho, B. | Testosterone Replacement, Muscle Strength, and Physical Function | Physical activity | No PA outcome |
| 2018 | Nyman, S. R.; Adamczewska, N.; Howlett, N. | Systematic review of behaviour change techniques to promote participation in physical activity among people with dementia | Physical activity | No PA outcome |
| 2018 | Rhodes, Ryan E.; Saelens, Brian E.; Sauvage-Mar, Claire | Understanding Physical Activity through Interactions Between the Built Environment and Social Cognition: A Systematic Review | Age | could not confirm age |
| 2018 | Schulz, Maike; Romppel, Matthias; Grande, Gesine | Built environment and health: a systematic review of studies in Germany | Age | could not confirm age |
| 2018 | Sharara, E. et al. | Physical inactivity, gender and culture in Arab countries: a systematic assessment of the literature | Age | Wrong age group |
| 2018 | Shishehgar, M.; Kerr, D.; Blake, J. | A systematic review of research into how robotic technology can help older people | Physical activity | No physical activity outcome |
| 2018 | Torres-de Araujo, J. R. et al. | Functional, nutritional and social factors associated with mobility limitations in the elderly: a systematic review | Physical activity | No PA outcome |
| 2018 | Tovar, Marlene; Walker, Janiece L.; Rew, Lynn | Factors Associated With Physical Activity in Latina Women: A Systematic Review | Age | Wrong age group |
| 2018 | Valenzuela, T et al. | Adherence to Technology-Based Exercise Programs in Older Adults: A Systematic Review | Determinant | Effectiveness |
| 2018 | Vseteckova, J. et al. | Barriers and facilitators to adherence to group exercise in institutionalized older people living with dementia: a systematic review | Physical activity | No PA outcome |
| 2018 | Woodbridge, R. et al. | Use of the physical environment to support everyday activities for people with dementia: A systematic review | Physical activity | No PA outcome |
| 2018 | Zusman, E. Z. et al. | A systematic review of evidence for older adults' sedentary behavior and physical activity after hip fracture | Physical activity | No physical activity outcome |
| 2019 | Alhaboby, Zhraa A. et al. | Cyber-Victimization of People With Chronic Conditions and Disabilities: A Systematic Review of Scope and Impact | Age | Wrong age group |
| 2019 | Alharbi, M. et al. | Data management and wearables in older adults: A systematic review | Physical activity | No physical activity outcome |
| 2019 | Alkaid Albqoor, Maha et al. | Systematic review: Self‚Äêrated health of Arab immigrants in the United States | Physical activity | No physical activity outcome |
| 2019 | An, R. et al. | Impact of ambient air pollution on physical activity and sedentary behavior in China: A systematic review | Age | could not extract older adults |
| 2019 | Arbel, Ifah; Bingham, Kathleen S.; Dawson, Deirdre R. | A Scoping Review of Literature on Sex and Gender Differences Among Dementia Spousal Caregivers | Physical activity | No PA outcome |
| 2019 | Baranowski, T.; and Lyons, E. J. | Scoping Review of Pokemon Go: Comprehensive Assessment of Augmented Reality for Physical Activity Change | Age | could not extract older adults |
| 2019 | Bouzas, C.; Bibiloni, M. D. M.; Tur, J. A. | Relationship between Body Image and Body Weight Control in Overweight >=55-Year-Old Adults: A Systematic Review | Physical activity | No PA outcome |
| 2019 | Brodin, N.; and Swardh, E. | Perceptions of physical activity in individuals with RA-a qualitative thematic synthesis | Unable to locate full text |  |
| 2019 | Coughlin, S. S. et al. | Survivorship issues in older breast cancer survivors | Determinant | reverse relationship |
| 2019 | Davergne, Thomas et al. | Use of Wearable Activity Trackers to Improve Physical Activity Behavior in Patients With Rheumatic and Musculoskeletal Diseases: A Systematic Review and Meta-Analysis | Age | could not extract older adults |
| 2019 | de Souza, I. M. B. et al. | Prevalence of low back pain in the elderly population: a systematic review | Physical activity | No physical activity outcome |
| 2019 | Eynon, M. et al. | Assessing the psychosocial factors associated with adherence to exercise referral schemes: A systematic review | Age | Wrong age group |
| 2019 | Finnegan, S.; Bruce, J.; Seers, K. | What enables older people to continue with their falls prevention exercises? A qualitative systematic review | Physical activity | No PA outcome |
| 2019 | Gee, Nancy R.; and Mueller, Megan K. | A Systematic Review of Research on Pet Ownership and Animal Interactions among Older Adults | Age | could not extract older adults |
| 2019 | Gough, C. et al. | Location monitoring of physical activity and participation in community dwelling older people: a scoping review | Age | Wrong age group |
| 2019 | Hassan, Suzan et al. | Factors prospectively associated with physical activity and dietary related outcomes in people with severe mental illness: A systematic review of longitudinal studies | Age | Wrong age group |
| 2019 | Hu, Y. L. et al. | Evidence to Improve Physical Activity among Medically Underserved Older adults: A Scoping Review | Age | could not confirm age |
| 2019 | Hughes, M et al. | Companion Animals and Health in Older Populations: A Systematic Review | Setting | Could not confirm community-dwelling |
| 2019 | Ige-Elegbede, J.; Pilkington, P.; Gray, S.; Powell, J. | Barriers and facilitators of physical activity among adults and older adults from Black and Minority Ethnic groups in the UK: A systematic review of qualitative studies | Physical activity | No PA outcome |
| 2019 | Jing, Zhang; and Bennett, Paul N. | The perception of people with chronic kidney disease towards exercise and physical activity: a literature review | Age | could not confirm age |
| 2019 | Josaphat, K. J. et al. | Use of Active Workstations in Individuals with Overweight or Obesity: A Systematic Review | Age | Wrong age group |
| 2019 | Kahan, D. | Critical appraisal of qualitative studies of muslim females' perceptions of physical activity barriers and facilitators | Wrong study design |  |
| 2019 | Kappen, Dennis L.; Mirza-Babaei, Pejman; Nacke, Lennart E. | Older adults' physical activity and exergames: A systematic review | Physical activity | No PA outcome |
| 2019 | Karsten, M. et al. | Is frailty associated with functional capacity decline in chronic cardiovascular disease patients? A systematic review and meta-analysis | Unable to locate full text |  |
| 2019 | Liangruenrom, N et al. | Correlates of physical activity and sedentary behaviour in the Thai population: a systematic review | Age | Could not extract older adults – Author was contacted |
| 2019 | Lounassalo, I. et al. | Distinct trajectories of physical activity and related factors during the life course in the general population: a systematic review | Age | could not extract older adults |
| 2019 | Maurer, C. et al. | Attitudes and needs of residents in long-term care facilities regarding physical activity-A systematic review and synthesis of qualitative studies | Physical activity | No PA outcome |
| 2019 | Miller, Matthew J. et al. | Factors influencing participation in physical activity after dysvascular amputation: a qualitative meta-synthesis | Physical activity | No PA outcome |
| 2019 | Mills, K. et al. | Minimal change in physical activity after lower limb joint arthroplasty, but the outcome measure may be contributing to the problem: a systematic review and meta-analysis | Determinant | not looking at |
| 2019 | Morgan, G. S. et al. | A life fulfilled: positively influencing physical activity in older adults - a systematic review and meta-ethnography | Physical activity | No PA outcome |
| 2019 | Rachele, J. N. et al. | Neighbourhood built environment and physical function among mid-to-older aged adults: A systematic review | Physical activity | No PA outcome |
| 2019 | Resurreccion, D. M. et al. | Factors associated with non-participation in and dropout from cardiac rehabilitation programmes: a systematic review of prospective cohort studies | Physical activity | No PA outcome |
| 2019 | Riggs, Donna M.; and Killingback, Clare | What factors influence physical activity participation in people with rheumatoid arthritis? | Age | Wrong age group |
| 2019 | Sattar, S. et al. | Perception of Structured Exercise Programs and Factors Associated with Participation and Adherence among Men with Prostate Cancer: A Scoping Review | Unable to locate full text |  |
| 2019 | Spiteri, K. et al. | Barriers and Motivators of Physical Activity Participation in Middle-aged and Older-adults - A Systematic Review | Physical activity | No PA outcome |
| 2019 | Tollosa, D. N. et al. | Adherence to multiple health behaviours in cancer survivors: a systematic review and meta-analysis | Determinant | not looking at |
| 2019 | Vierula, M.; Pilon, S.; McIsaac, D. I. | Systematic review of predictors of adherence to exercise therapy in older adults with a medical condition | Unable to locate full text |  |
| 2019 | Wallis, J. A. et al. | Experience of living with knee osteoarthritis: A systematic review of qualitative studies | Physical activity | No PA outcome |
| 2019 | Wang, D. X. M. et al. | Muscle mass, strength, and physical performance predicting activities of daily living: a meta-analysis | Duplicate |  |
| 2019 | Yarmohammadi, S et al. | A systematic review of barriers and motivators to physical activity in aged adults in Iran and worldwide | Physical activity | No PA outcome |
| 2020 | Aggarwal, B.; Xiong, Q.; Schroeder-Butterfill, E. | Impact of the use of the internet on quality of life in older adults: review of literature | Physical activity | No PA outcome |
| 2020 | Baranowski, T.; and Lyons, E. J. | Scoping Review of Pokemon Go: Comprehensive Assessment of Augmented Reality for Physical Activity Change | Age | Wrong age group |
| 2020 | Barr, A. L. et al. | Sociodemographic inequities associated with participation in leisure-time physical activity in sub-Saharan Africa: an individual participant data meta-analysis | Age | Wrong age group |
| 2020 | Bosma, M. S. et al. | Impact of visuospatial neglect post-stroke on daily activities, participation and informal caregiver burden: A systematic review | Physical activity | No PA outcome |
| 2020 | Bourne, J. E. et al. | The impact of e-cycling on travel behaviour: A scoping review | Age | Wrong age group |
| 2020 | Burse, N. R. et al. | Physical activity barriers and resources among black women with a history of breast and endometrial cancer: a systematic review | Age | Wrong age group |
| 2020 | Caputo, E. L.; and Reichert, F. F. | Studies of Physical Activity and COVID-19 During the Pandemic: A Scoping Review | Age | could not extract older adults |
| 2020 | Castrillon, C. I. M. et al. | Are people in the bush really physically active? A systematic review and meta-analysis of physical activity and sedentary behaviour in rural Australians populations | Age | Wrong age group |
| 2020 | Di Lorito, C et al. | Adherence to exercise interventions in older people with mild cognitive impairment and dementia: A systematic review and meta-analysis | Setting | Not specified |
| 2020 | Fritz, H. et al. | Neighborhood Characteristics and Frailty: A Scoping Review | Physical activity | No PA outcome |
| 2020 | Goethals, L. et al. | Social marketing interventions to promote physical activity among 60 years and older: a systematic review of the literature | Determinant | effectiveness |
| 2020 | Graham, Hilary et al. | Older people's experiences of everyday travel in the urban environment: a thematic synthesis of qualitative studies in the United Kingdom | Determinant | not looking at |
| 2020 | Gropper, H. et al. | The impact of life events and transitions on physical activity: A scoping review | Age | could not extract older adults |
| 2020 | Harvey, K.; and Griffin, M. | Exercise Instructors for Older Adult Fitness: A Review of the Literature | Age | Wrong age group |
| 2020 | Jimenez-Zazo, F. et al. | Transtheoretical Model for Physical Activity in Older Adults: Systematic Review | Determinant | not looking at |
| 2020 | John, J. M.; Haug, V.; Thiel, A. | Physical Activity Behavior from a Transdisciplinary Biopsychosocial Perspective: a Scoping Review | Age | Wrong age group |
| 2020 | Kiernan, S. et al. | Physical functioning limitations and physical activity of people experiencing homelessness: A scoping review | Age | Wrong age group |
| 2020 | Kompf, J. | Implementation Intentions for Exercise and Physical Activity: Who Do They Work For? A Systematic Review | Age | Wrong age group |
| 2020 | Liu, S. H. et al. | Physical activity and attitudes and perceptions towards physical activity in patients with spondyloarthritis: A systematic review | Age | Wrong age group |
| 2020 | McHale, S. et al. | A systematic review and thematic synthesis exploring how a previous experience of physical activity influences engagement with cardiac rehabilitation | Physical activity | No PA outcome |
| 2020 | Peters, M. et al. | Measuring the association of objective and perceived neighborhood environment with physical activity in older adults: challenges and implications from a systematic review | Determinant | not looking at |
| 2020 | Qin, W.; Blanchette, J. E.; Yoon, M. | Self-Efficacy and Diabetes Self-Management in Middle-Aged and Older Adults in the United States: A Systematic Review | Age | could not extract older adults |
| 2020 | Rigby, B. P.; Dodd-Reynolds, C. J.; Oliver, E. J. | Inequities and inequalities in outdoor walking groups: A scoping review | Determinant | not looking at |
| 2020 | Stenner, B. J.; Buckley, J. D.; Mosewich, A. D. | Reasons why older adults play sport: A systematic review | Physical activity | No PA outcome |
| 2020 | Vseteckova, J. et al. | Barriers and facilitators to adherence to walking group exercise in older people living with dementia in the community: a systematic review | Age | could not confirm age |
| 2020 | Wang, D. X. M. et al. | Muscle mass, strength, and physical performance predicting activities of daily living: a meta-analysis | Physical activity | No PA outcome |
| 2020 | Wang, Ya-Ling; Hou, Huei-Tse; Tsai, Chin-Chung | A systematic literature review of the impacts of digital games designed for older adults | Physical activity | No PA outcome |
| 2020 | Zhen, X. et al. | Modifiable facilitators and barriers to exercise adherence in older adults with MCI/dementia using the Theoretical Domains Framework: a systematic review protocol | Wrong study design |  |
| 2020 | Zhong, S et al. | Intergenerational communities: A systematic literature review of intergenerational interactions and older adults' health-related outcomes | Setting | Not specified |
| 2021 | Adeloye, D. et al. | Epidemiology of physical inactivity in Nigeria: a systematic review and meta-analysis | Age | Wrong age group |
| 2021 | Alaazi, D. A.; Menon, D.; Stafinski, T. | Health, quality of life, and wellbeing of older slum dwellers in sub-Saharan Africa: A scoping review | Physical activity | No PA outcome |
| 2021 | Allen, B. et al. | Facilitators and Barriers to Physical Activity and Sport Participation Experienced by Aboriginal and Torres Strait Islander Adults: A Mixed Method Review | Age | Wrong age group |
| 2021 | Baker, N.; Lawn, S.; Gordon, S. J.; George, S. | Older Adults' Experiences of Goals in Health: A Systematic Review and Metasynthesis | Physical activity | No PA outcome |
| 2021 | Bernard, P. et al. | Climate Change, Physical Activity and Sport: A Systematic Review | Age | could not confirm age |
| 2021 | Boey, D. et al. | The impact of low vision on activities, participation, and goals among older adults: a scoping review | Physical activity | No PA outcome |
| 2021 | Cannella, V.; Villar, F.; Serrat, R.; Tulle, E. | Psychosocial Aspects of Participation in Competitive Sports Among Older Athletes: A Scoping Review | Age | Wrong age group |
| 2021 | Chair, S. Y.; and Zou, H.; Cao, X. | A systematic review of effects of recorded music listening during exercise on physical activity adherence and health outcomes in patients with coronary heart disease | Determinant | effectiveness |
| 2021 | Elisabeth, A. L.; Karlen, S. B.; Magkos, F. | The Effect of COVID-19-related Lockdowns on Diet and Physical Activity in Older Adults: A Systematic Review | Age | could not extract older adults |
| 2021 | Engels, C. et al. | Leisure and Productivity in Older Adults with Cancer: A Systematic Review | Determinant | not looking at |
| 2021 | Gaertner, B. et al. | Older people at the beginning of the COVID-19 pandemic: A scoping review | Physical activity | No PA outcome |
| 2021 | Gorska, S. et al. | A systematic review and correlational meta-analysis of factors associated with resilience of normally aging, community-living older adults | Physical activity | No PA outcome |
| 2021 | Gough, C. et al. | Location monitoring of physical activity and participation in community dwelling older people: a scoping review | Physical activity | No PA outcome |
| 2021 | Horne, J. et al. | A Systematic Review on the Prevalence of Physical Activity, and Barriers and Facilitators to Physical Activity, in Informal Carers in the United Kingdom | Physical activity | No PA outcome |
| 2021 | Hu, R. X.; Luo, M.; Zhang, A.; Li, L. W. | Associations of Ageism and Health: A Systematic Review of Quantitative Observational Studies | Physical activity | No PA outcome |
| 2021 | Huffman, M. K. et al. | Maintenance motives for physical activity among older adults: a systematic review and meta-analysis | Age | Wrong age group |
| 2021 | Kendrick, D. et al. | Older gay men's engagement with physical activity: A scoping review | Physical activity | No PA outcome |
| 2021 | Kim, Y. B. et al. | Ambient air pollution and movement behaviours: A scoping review | Age | Wrong age group |
| 2021 | Knight, R. L. et al. | Moving Forward: Understanding Correlates of Physical Activity and Sedentary Behaviour during COVID-19-An Integrative Review and Socioecological Approach | Age | Wrong age group |
| 2021 | Lebrasseur, A. et al. | Impact of the COVID-19 Pandemic on Older Adults: Rapid Review | Wrong study design |  |
| 2021 | Lim, M. L. et al. | Association between health literacy and physical activity in older people: a systematic review and meta-analysis | Age | Wrong age group |
| 2021 | Marques, A. et al. | Bidirectional Association between Physical Activity and Dopamine Across Adulthood-A Systematic Review | Determinant | not looking at |
| 2021 | Martins, L. C. G. et al. | The factors related to a sedentary lifestyle: A meta-analysis review | Physical activity | No PA outcome |
| 2021 | McLaughlin, M. et al. | Associations Between Digital Health Intervention Engagement, Physical Activity, and Sedentary Behavior: Systematic Review and Meta-analysis | Age | Wrong age group |
| 2021 | Moore, A. et al. | Volunteer Impact on Health-Related Outcomes for Seniors: a Systematic Review And Meta-Analysis | Determinant | effectiveness |
| 2021 | Ng, Y. L. et al. | Effectiveness of Outdoor Exercise Parks on Health Outcomes in Older Adults-A Mixed-Methods Systematic Review and Meta-Analysis | Determinant | not looking at |
| 2021 | Pereira, H. V. et al. | Systematic Review of Psychological and Behavioral Correlates of Recreational Running | Age | could not extract older adults |
| 2021 | Remm, S. et al. | Understanding relationships between general self-efficacy and the healthy ageing of older people: An integrative review | Physical activity | No PA outcome |
| 2021 | Rivera-Torres, S. et al. | Older Adults' Mental Health Through Leisure Activities During COVID-19: A Scoping Review | Determinant | reverse relationship |
| 2021 | Roordink, E. M. et al. | Predictors of lapse and relapse in physical activity and dietary behaviour: a systematic search and review on prospective studies | Age | Wrong age group |
| 2021 | Scott, J. et al. | A systematic review of the physical activity levels of acutely ill older adults in Hospital At Home settings: an under-researched field | Determinant | not looking at |
| 2021 | Shahbazi, M. et al. | The opportunities and challenges of using mobile health in elderly self-care | Physical activity | No PA outcome |
| 2021 | Sindel, D.; and Oral, A. | Are environmental and behavioral interventions effective in reducing physical activity limitation and preventing falls in the visually impaired older individuals? - A Cochrane Review summary with commentary | Determinant | effectiveness |
| 2021 | Torossian, M.; and Jacelon, C. S. | Chronic Illness and Fatigue in Older Individuals: A Systematic Review | Determinant | not looking at |
| 2021 | Vancampfort, D. et al. | Dropout from exercise randomized controlled trials among people with anxiety and stress-related disorders: A meta-analysis and meta-regression | Age | Wrong age group |
| 2021 | Vancampfort, D. et al. | Physical activity correlates in children and adolescents, adults, and older adults with an intellectual disability: a systematic review | Age | Wrong age group |
| 2021 | Vancampfort, D.; and Ward, P. B. | Physical activity correlates across the lifespan in people with epilepsy: a systematic review | Age | Wrong age group |
| 2022 | Alfatafta, H. et al. | Effect of the knee replacement surgery on activity level based on ActivPAL: a systematic review and meta-analysis study | Determinant | not looking at |
| 2022 | Ali, S.; Kennedy, M.; Salma, J. | A Scoping Review on Community-Based Programs to Promote Physical Activity in Older Immigrants | Physical activity | No PA outcome |
| 2022 | Alpalhao, V.; Cordeiro, N.; Pezarat-Correia, P. | Kinesiophobia and Fear Avoidance in Older Adults: A Scoping Review on the State of Research Activity | Physical activity | No PA outcome |
| 2022 | Auais, M. et al. | Understanding the relationship between psychological factors and important health outcomes in older adults with hip fracture: A structured scoping review | Physical activity | No PA outcome |
| 2022 | Azim, F et al. | Indoor Built Environment and Older Adults' Activity: A Systematic Review | Setting | Less than 80% community-dwelling |
| 2022 | Bhatia, D. et al. | Outdoor Community Ambulation Interventions to Improve Physical and Mental Health in Older Adults: A Systematic Review and Meta-Analysis | Determinant | effectiveness |
| 2022 | Buckley, T. D. | A Scoping Review of Psychological Sense of Community among Community-Dwelling Older Adults | Physical activity | No PA outcome |
| 2022 | Cataldi, S. et al. | The Relationship between Physical Activity, Physical Exercise, and Human Gut Microbiota in Healthy and Unhealthy Subjects: A Systematic Review | Age | Wrong age group |
| 2022 | Chen, P. W. et al. | Productive Aging by Environmental Volunteerism: A Systematic Review | Physical activity | No PA outcome |
| 2022 | Christensen, A.; Bond, S.; McKenna, J. | The COVID-19 Conundrum: Keeping safe while becoming inactive. A rapid review of physical activity, sedentary behaviour, and exercise in adults by gender and age | Age | could not extract older adults |
| 2022 | Delpino, F. M. et al. | Physical Activity and Multimorbidity Among Community-Dwelling Older Adults: A Systematic Review With Meta-Analysis | Determinant | reverse relationship |
| 2022 | Gidgup, M. J. R. et al. | Barriers and Enablers to Older Indigenous People Engaging in Physical Activity-A Qualitative Systematic Review | Age | Wrong age group |
| 2022 | Kalu, M. E. et al. | Cognitive, psychological and social factors associated with older adults' mobility: a scoping review of self-report and performance-based measures | Physical activity | No PA outcome |
| 2022 | Ng, L.; Oliver, E.; Laver, K. | Beyond garden design: A review of outdoor occupation in hospital and residential care settings for people with dementia | Determinant | reverse relationship |
| 2022 | Ning, Y. et al. | Barriers and facilitators to physical activity participation in patients with head and neck cancer: a scoping review | Age | Wrong age group |
| 2022 | Noh, W.; and Kim, K. Y. | Review of Ecological Approach Factors Affecting Physical Activity among Older People | Setting | Not specified |
| 2022 | Oliveira, M.R et al. | Covid-19 and the impact on the physical activity level of elderly people: A systematic review | Setting | Not specified |
| 2022 | Padeiro, M. et al. | Neighborhood Attributes and Well-Being Among Older Adults in Urban Areas: A Mixed-Methods Systematic Review | Physical activity | No PA outcome |
| 2022 | Panicker, R. M. and Chandrasekaran, B. | "Wearables on vogue": a scoping review on wearables on physical activity and sedentary behavior during COVID-19 pandemic | Age | could not confirm age |
| 2022 | Pelletier, C et al. | Physical activity promotion in rural health care settings: A rapid realist review | Age | could not extract older adults |
| 2022 | Shaw, J et al. | Predictors of adherence to prescribed exercise programs for older adults with medical or surgical indications for exercise: a systematic review | Setting | Not specified |
| 2022 | Schmidt, L. L. et al. | Social Interaction and Physical Activity Among Rural Older Adults: A Scoping Review | Physical activity | No PA outcome |
| 2022 | Silveira, E. A. et al. | Sedentary behavior, physical inactivity, abdominal obesity and obesity in adults and older adults: A systematic review and meta-analysis | Age | could not extract older adults |
| 2022 | Smith, L. et al. | Physical activity and visual difficulties in 36 low- and middle-income countries | Wrong study design |  |
| 2022 | Su, Y. et al. | Fatigue in community-dwelling older adults: A review of definitions, measures, and related factors | Determinant | reverse relationship |
| 2022 | Vasudevan, A. and Ford, E. | Motivational Factors and Barriers Towards Initiating and Maintaining Strength Training in Women: a Systematic Review and Meta-synthesis | Age | Wrong age group |
| 2022 | Wunsch, K.; Kienberger, K.; Niessner, C. | Changes in Physical Activity Patterns Due to the Covid-19 Pandemic: A Systematic Review and Meta-Analysis | Setting | Less than 80% community-dwelling |
| 2022 | Xie, L. et al. | Electronic health literacy and health-related outcomes among older adults: A systematic review | Physical activity | No PA outcome |
| 2022 | Yang, Y. et al. | Measurement of Adherence to mHealth Physical Activity Interventions and Exploration of the Factors That Affect the Adherence: Scoping Review and Proposed Framework | Age | could not extract older adults |
